# Supplementary material for: An airway organoid-based screen identifies a role for the HIF1α-glycolysis axis in SARS-CoV-2 infection
Source: Cell Rep. 2021 Oct 15;37(6):109920. doi: 10.1016/j.celrep.2021.109920 (PMC8516798; doi:10.1016/j.celrep.2021.109920)
Supplement: Document S1. Figures S1–S4 and Tables S3, S5, and S6 [file mmc1.pdf]

Cell Reports, Volume *37*

Supplemental information

An airway organoid-based screen identifies a role for the HIF1α-glycolysis axis in SARS-CoV-2 infection

Xiaohua Duan, Xuming Tang, Manoj S. Nair, Tuo Zhang, Yunping Qiu, Wei Zhang, Pengfei Wang, Yaoxing Huang, Jenny Xiang, Hui Wang, Robert E. Schwartz, David D. Ho, Todd Evans, and Shuibing Chen

Figure S1

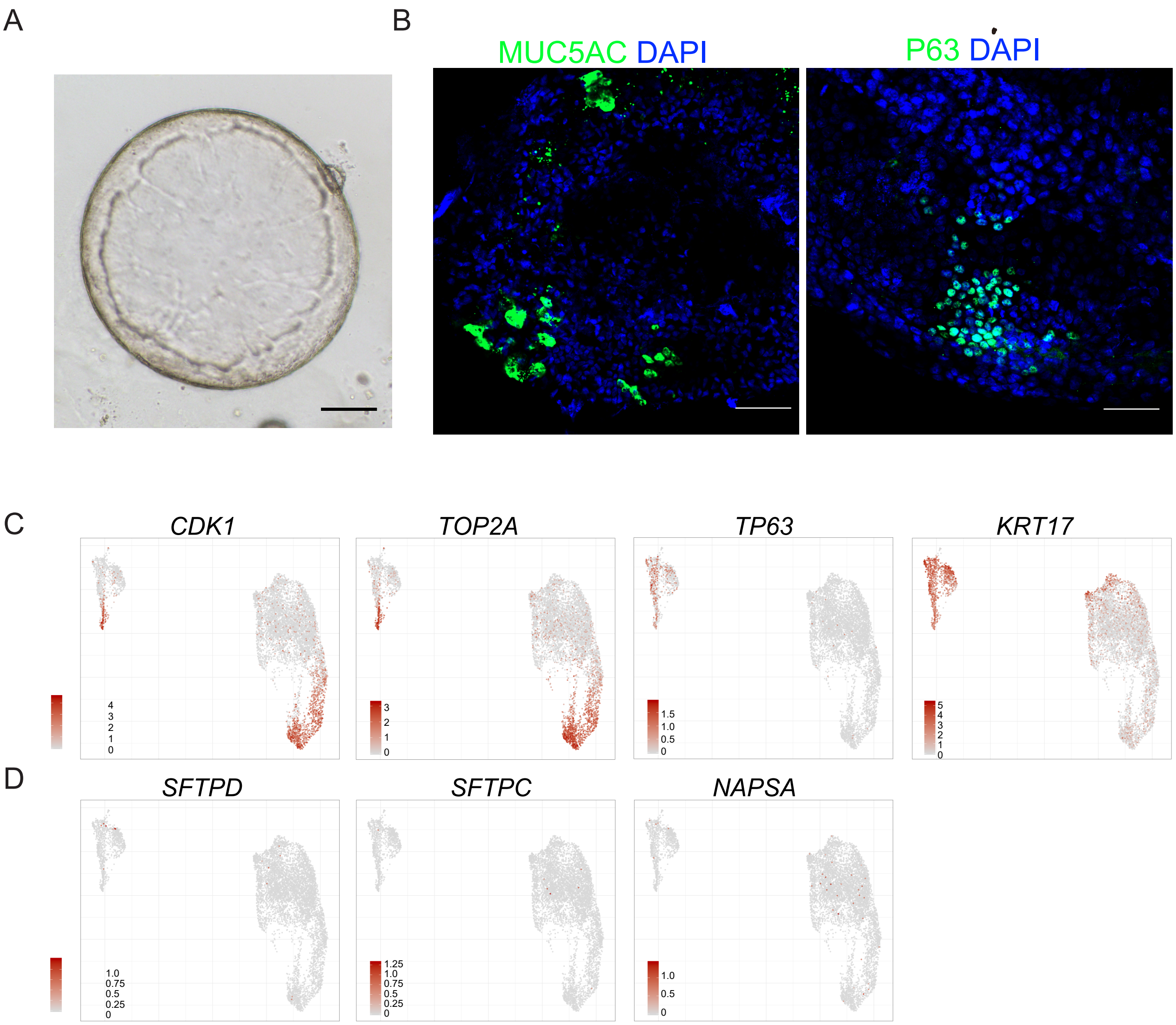
A
B
MUC5AC DAPI
P63 DAPI
C
CDK1
TOP2A
TP63
KRT17
4
3
2
1
0
3
2
1
0
1.5
1.0
0.5
0
5
4
3
2
1
0
D
SFTPD
SFTPC
NAPSA
1.0
0.75
0.5
0.25
0
1.25
1.0
0.75
0.5
0.25
0
1.0
0.5
0

Figure S1. Derivation and Characterization of hPSC-AOs.

(A) Phase contrast image of a representative hPSC-ASs. Scale bar = 400 μm.

(B) Representative confocal images of goblet cell marker MUC5AC and basal cell marker P63 in hPSC-AOs. DAPI stains nuclei. Scale bar = 100 μm.

(C and **D)** UMAP showing the expression levels of (C) hPSC-AOs cell markers and (D) alveolar epithelial type 2 cell markers**.** Related to Figure 1.

Figure S2

A

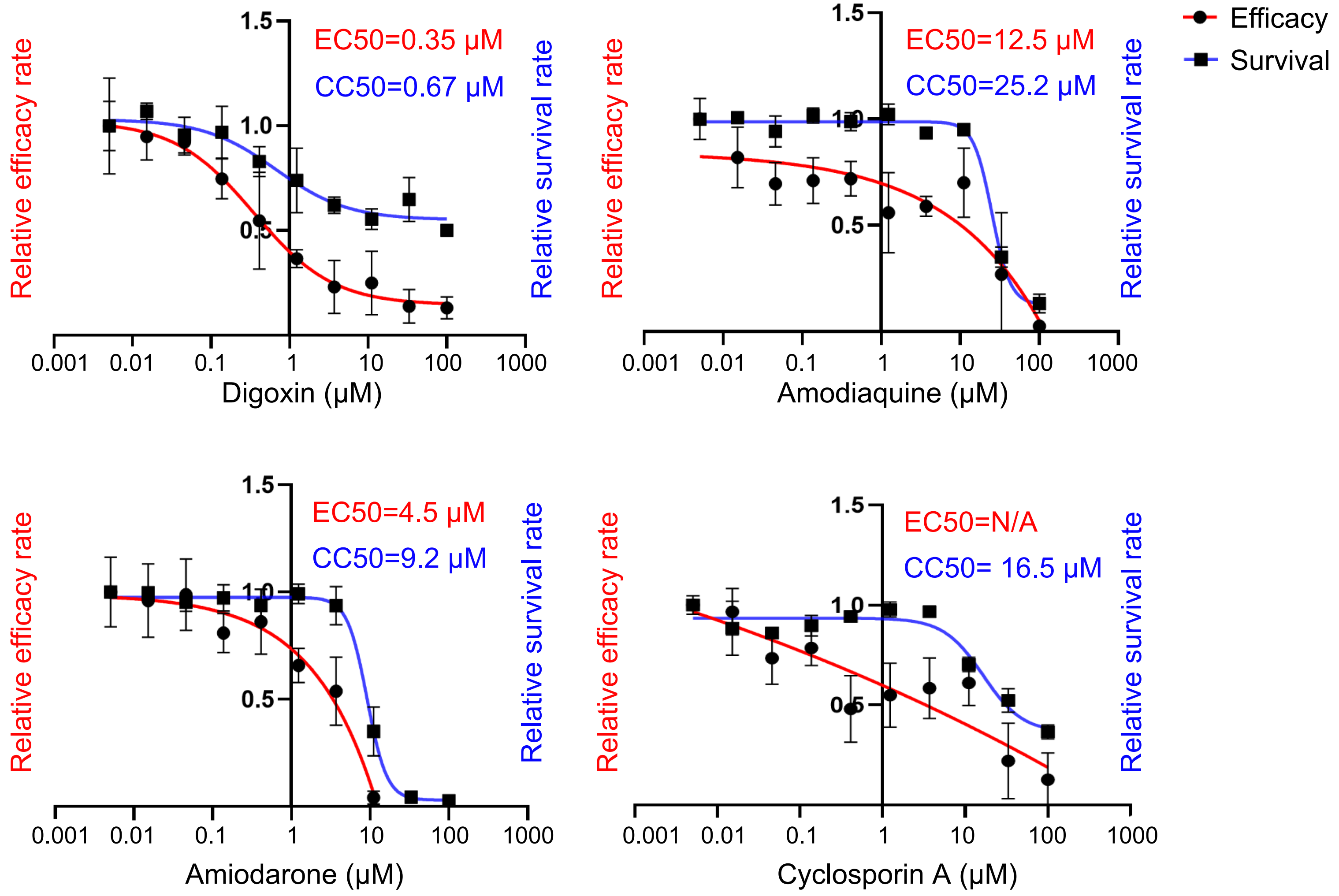
Efficacy
Survival
EC50=0.35 μM
CC50=0.67 μM
Relative efficacy rate
Relative survival rate
Digoxin (μM)
EC50=12.5 μM
CC50=25.2 μM
Amodiaquine (μM)
EC50=4.5 μM
CC50=9.2 μM
Amiodarone (μM)
EC50=N/A
CC50= 16.5 μM
Cyclosporin A (μM)
0.001
0.01
0.1
1
10
100
1000
1.5
1.0
0.5

B

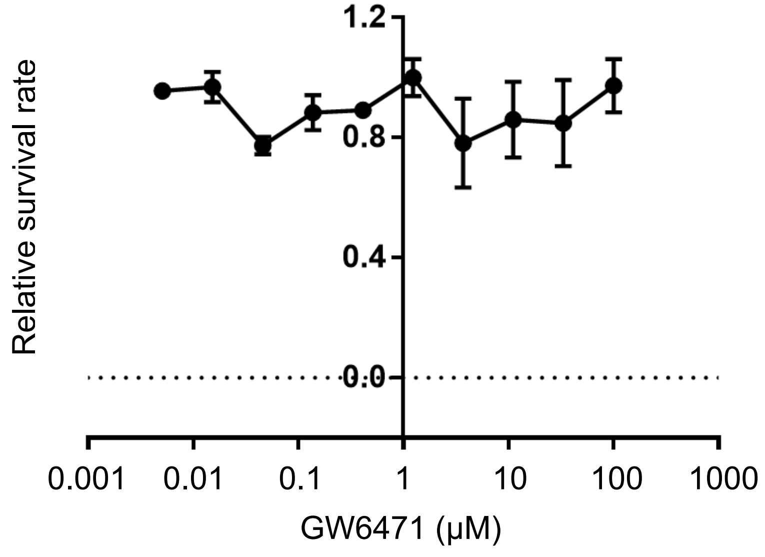
Relative survival rate
1.2
0.8
0.4
0.0
0.001
0.01
0.1
1
10
100
1000
GW6471 (μM)

Figure S2. Confirmation of hit compounds from high content screen.

(A) Efficacy and toxicity curves of hit compounds identified from the high content screen.

(B) Cell survival curve of GW6471 on hPSC-AOs.

Data is presented as mean ± SEM. N=3 biological replicates. Related to Figure 3.

Figure S3

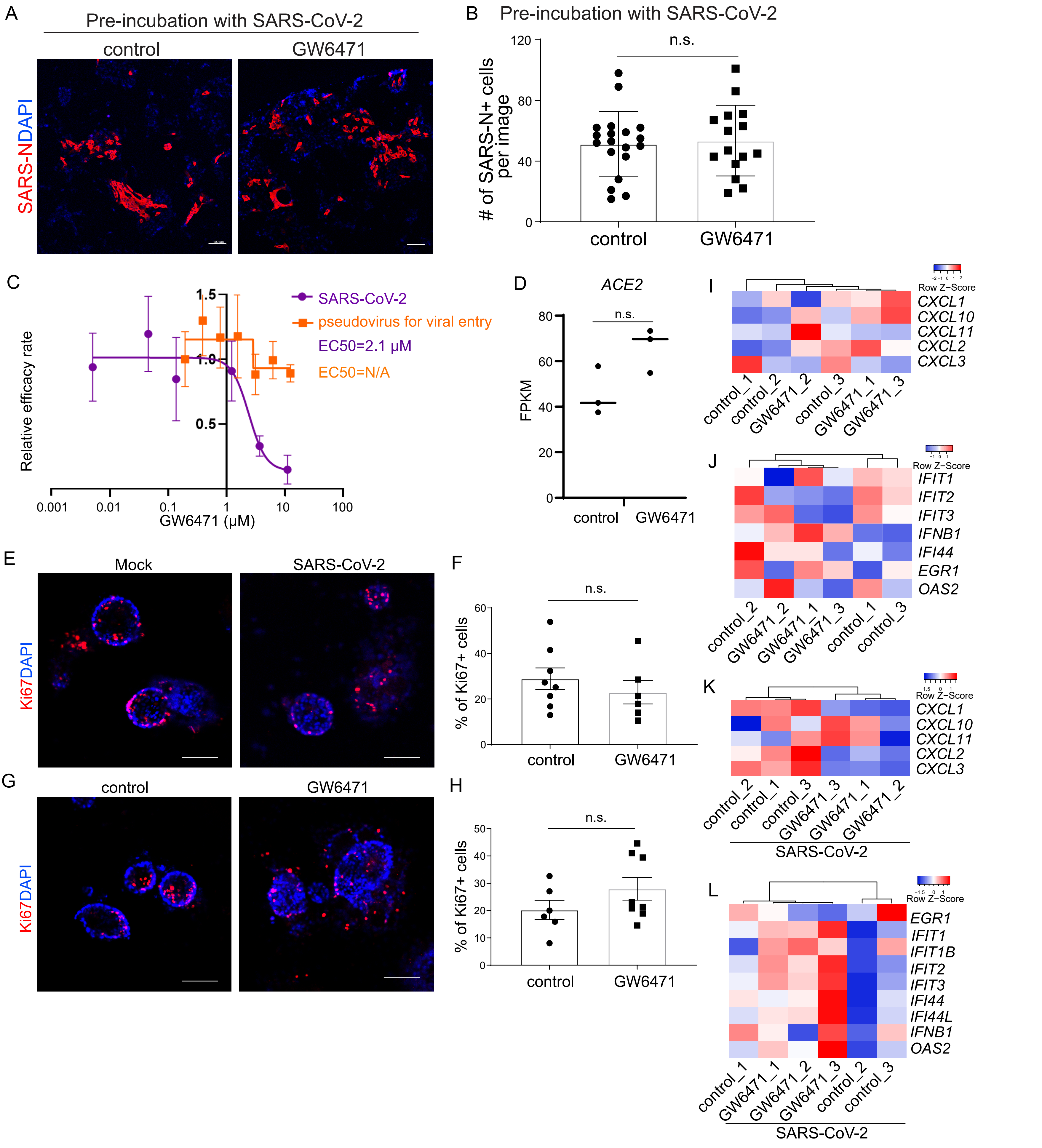
A
Pre-incubation with SARS-CoV-2
control
GW6471
SARS-N DAPI
B
Pre-incubation with SARS-CoV-2
n.s.
# of SARS-N+ cells per image
120
80
40
0
control
GW6471
C
SARS-CoV-2
pseudovirus for viral entry
EC50=2.1 μM
EC50=N/A
Relative efficacy rate
1.5
1.0
0.5
0.001
0.01
0.1
1
10
100
GW6471 (μM)
D
ACE2
n.s.
FPKM
80
60
40
20
0
control
GW6471
I
Row Z-Score
CXCL1
CXCL10
CXCL11
CXCL2
CXCL3
control_1
control_2
GW6471_2
control_3
GW6471_1
GW6471_3
J
Row Z-Score
IFIT1
IFIT2
IFIT3
IFNB1
IFI44
EGR1
OAS2
control_2
GW6471_2
GW6471_1
GW6471_3
control_1
control_3
E
Mock
SARS-CoV-2
Ki67 DAPI
F
n.s.
% of Ki67+ cells
60
40
20
0
control
GW6471
K
Row Z-Score
CXCL1
CXCL10
CXCL11
CXCL2
CXCL3
control_2
control_1
control_3
GW6471_3
GW6471_1
GW6471_2
SARS-CoV-2
G
control
GW6471
Ki67 DAPI
H
n.s.
% of Ki67+ cells
50
40
30
20
10
0
control
GW6471
L
Row Z-Score
EGR1
IFIT1
IFIT1B
IFIT2
IFIT3
IFI44
IFI44L
IFNB1
OAS2
control_1
GW6471_1
GW6471_2
GW6471_3
control_2
control_3
SARS-CoV-2

Figure S3. The impact of GW6471 on viral cycle and host AOs.

(A and **B)** Representative confocal images (A) and quantification (B) at 48 hpi of SARS-N$^+$ cells of hPSC-AOs, infected with SARS-CoV-2 which was pre-incubated with GW6471 or control (MOI=0.2). Scale bar = 100 μm. Data is presented as mean ± SEM. N=3 biological replicates.

(C) Efficacy curves of GW6471 on SARS-CoV-2 or pseudovirus. Data is presented as mean ± SD. N=3 biological replicates.

(D) FPKM value of ACE2 of control or 10 μM GW6471 treated hPSC-AOs at 48 hpi (MOI=0.2). Data is presented as mean ± SD. N=3 biological replicates.

(E and **F)** Representative confocal images (E) and quantification (F) of Ki67$^+$ cells of mock or SARS-CoV-2 infected (MOI=0.2) hPSC-AOs. Scale bar = 100 μm. Data is presented as mean ± SEM. N=3 biological replicates.

(G and **H)** Representative confocal images (G) and quantification (H) of Ki67$^+$ cells of GW6471 or control treated hPSC-AOs (MOI=0.2). Scale bar = 100 μm. Data is presented as mean ± SEM. N=3 biological replicates.

(I and **K)** Heatmap of chemokine (I) and interferon (K) expression of GW6471 or control treated hPSC-AOs in the absence of SARS-CoV-2.

(J and **L)** Heatmap of chemokine (J) and interferon (L) expression of GW6471 or control treated hPSC-AOs in the presence of SARS-CoV-2 (MOI=0.2).

P values were calculated by unpaired two-tailed Student's t test.

Related to Figure 4.

Figure S4

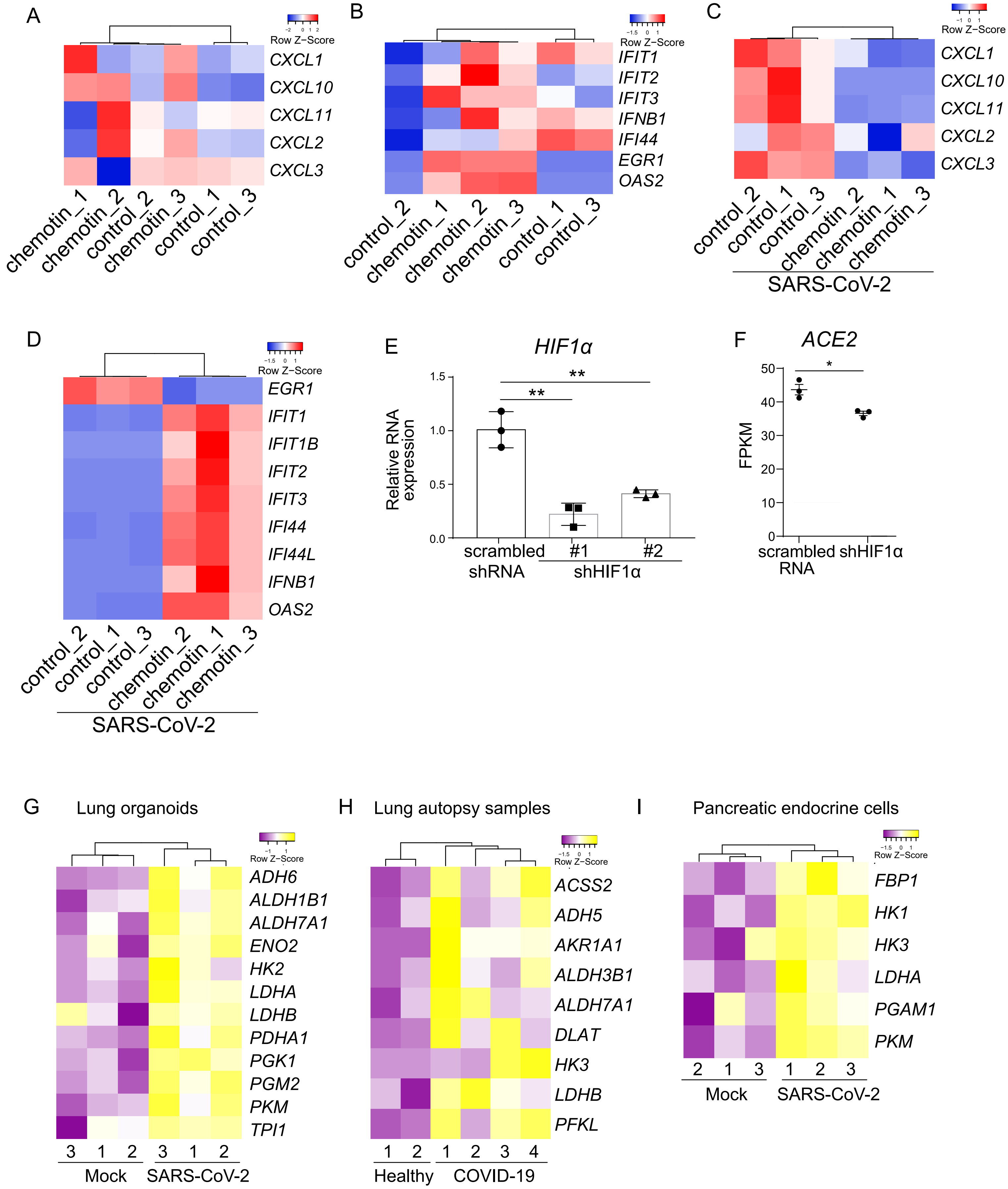
A
Row Z−Score
CXCL1
CXCL10
CXCL11
CXCL2
CXCL3
chemotin_1 chemotin_2 control_2 chemotin_3 control_1 control_3
B
Row Z−Score
IFIT1
IFIT2
IFIT3
IFNB1
IFI44
EGR1
OAS2
control_2 chemotin_1 chemotin_2 chemotin_3 control_1 control_3
C
Row Z−Score
CXCL1
CXCL10
CXCL11
CXCL2
CXCL3
control_2 control_1 control_3 chemotin_2 chemotin_1 chemotin_3
SARS-CoV-2
D
Row Z−Score
EGR1
IFIT1
IFIT1B
IFIT2
IFIT3
IFI44
IFI44L
IFNB1
OAS2
control_2 control_1 control_3 chemotin_2 chemotin_1 chemotin_3
SARS-CoV-2
E
HIF1α
Relative RNA expression
1.5
1.0
0.5
0.0
**
**
scrambled shRNA
#1
#2
shHIF1α
F
ACE2
FPKM
50
40
30
20
10
0
*
scrambled RNA
shHIF1α
G
Lung organoids
Row Z−Score
ADH6
ALDH1B1
ALDH7A1
ENO2
HK2
LDHA
LDHB
PDHA1
PGK1
PGM2
PKM
TPI1
3 1 2 3 1 2
Mock
SARS-CoV-2
H
Lung autopsy samples
Row Z−Score
ACSS2
ADH5
AKR1A1
ALDH3B1
ALDH7A1
DLAT
HK3
LDHB
PFKL
1 2 1 2 3 4
Healthy
COVID-19
I
Pancreatic endocrine cells
Row Z−Score
FBP1
HK1
HK3
LDHA
PGAM1
PKM
2 1 3 1 2 3
Mock
SARS-CoV-2

Figure S4. RNA-seq analysis.

(A and **B)** Heatmap of chemokine (A) and interferon (B) expression of chetomin or control treated hPSC-AOs in the absence of SARS-CoV-2.

(C and **D)** Heatmap of chemokine (C) and interferon (D) expression of chetomin or control treated hPSC-AOs in the presence of SARS-CoV-2 (MOI=0.2).

(E) Relative HIF1α mRNA expression in hPSC-AOs expressing shHIF1α or scrambled shRNA. Data is presented as mean ± SEM. N=3 biological replicates.

(F) FPKM value of ACE2 of control or chetomin-treated hPSC-AOs at 48 hpi (MOI=0.2). Data is presented as mean ± SD. N=3 biological replicates.

(**G**)Heatmap of glycolysis pathway associated genes in SARS-CoV-2 infected hPSC-lung organoids (GSE155241).

(**H**) Heatmap of glycolysis pathway associated genes in autopsy samples of COVID-19 patients (GSE155241).

(**I**) Heatmap of glycolysis pathway associated genes in SARS-CoV-2 infected pancreatic endocrine cells (GSE147903).

P values were calculated by unpaired two-tailed Student's t test. Related to Figure 4 and Figure 6.

Table S3. shRNAs for HIF1α knockdown. Related to Figure 4.

Scrambled shRNA	*GCACTACCAGAGCTAACTCAGATAGTACT*
shHIF1α_#1	*AGCTTGCTCATCAGTTGCCACTTCCACAT*
shHIF1α_#2	*TACGTTGTGAGTGGTATTATTCAGCACGA*

Table S5. Antibodies used for immunocytochemistry. Related to STAR*METHODS.

Usage	Antibody	Clone #	Host	Catalog #	Vendor	Dilution
Immunocytochemistry	Human ACE-2 Antibody	Polyclonal	Goat	AF933	R&D Systems	1:200
Immunocytochemistry	CDX2	CDX2-88	Mouse	MU392A-UC	Biogenex	1:500
Immunocytochemistry	Cytokeratin-20	SPM140	Mouse	sc-56522	Santa Cruz	1:100
Immunocytochemistry	FOXJ1	2A5	Mouse	14-9965-82	Thermo Fisher Scientific	1:100
Immunocytochemistry	acetyl-alpha tubulin	6-11B-1	Mouse	MABT868	Sigma-Aldrich	1:3000
Immunocytochemistry	acetyl-alpha tubulin	D20G3	Rabbit	5335	Cell Signaling	1:3000
Immunocytochemistry	MUC5AC	45M1	Mouse	MA5-12178	Thermo Fisher Scientific	1:100
Immunocytochemistry	P63	4A4	Mouse	CM163A	Biocare	1:100
Immunocytochemistry	SARS-CoV/SARS-CoV-2 Nucleocapsid Antibody	R001	Rabbit	40143-R001	Sino Biological	1:200
Immunocytochemistry	Ki67 Antibody	SP6	Rabbit	RM-9106-S1	Thermo Fisher Scientific	1:500
Immunocytochemistry	Donkey anti-Mouse IgG (H+L) Cross-Adsorbed Secondary Antibody, Alexa Fluor 488	Polyclonal	Donkey	#A-21202	Thermo Fisher Scientific	1:500

Immunocytochemistry	Donkey anti-Rabbit IgG (H+L) Secondary Antibody, Alexa Fluor 594	Polyclonal	Donkey	#A-21207	Thermo Fisher Scientific	1:500
Immunocytochemistry	Donkey anti-Goat IgG (H+L) Cross-Adsorbed Secondary Antibody, Alexa Fluor 647	Polyclonal	Donkey	#A-21447	Thermo Fisher Scientific	1:500
Immunocytochemistry	Donkey anti-Goat IgG Secondary Antibody, Alexa Fluor 594	Polyclonal	Donkey	A32816	Thermo Fisher	1:500
Immunocytochemistry	Donkey anti-Rabbit IgG Secondary Antibody, Alexa Fluor 647	Polyclonal	Donkey	A32795	Thermo Fisher	1:500

Table S6. Primers used for qRT-PCR. Related to STAR*METHODS.

**Primer name**	**Sequence**
ACTB-Forward	*CGTCACCAACTGGGACGACA*
ACTB-Reverse	*CTTCTCGCGGTTGGCCTTGG*
SARS-CoV-2-TRS-L	*CTCTTGTAGATCTGTTCTCTAAACGAAC*
SARS-CoV-2-TRS-N	*GGTCCACCAAACGTAATGCG*
HIF1α-Forward	*TATGAGCCAGAAGAACTTTTAGGC*
HIF1α-Reverse	*CACCTCTTTTGGCAAGCATCCTG*
