# Supplementary material for: An airway organoid-based screen identifies a role for the HIF1α-glycolysis axis in SARS-CoV-2 infection
Source: Cell Rep. 2021 Oct 15;37(6):109920. doi: 10.1016/j.celrep.2021.109920 (PMC8516798; doi:10.1016/j.celrep.2021.109920)
Supplement: Document S2. Article plus supplemental information [file mmc7.pdf]

Cell Reports

Article

An airway organoid-based screen identifies a role for the HIF1α-glycolysis axis in SARS-CoV-2 infection

Graphical abstract

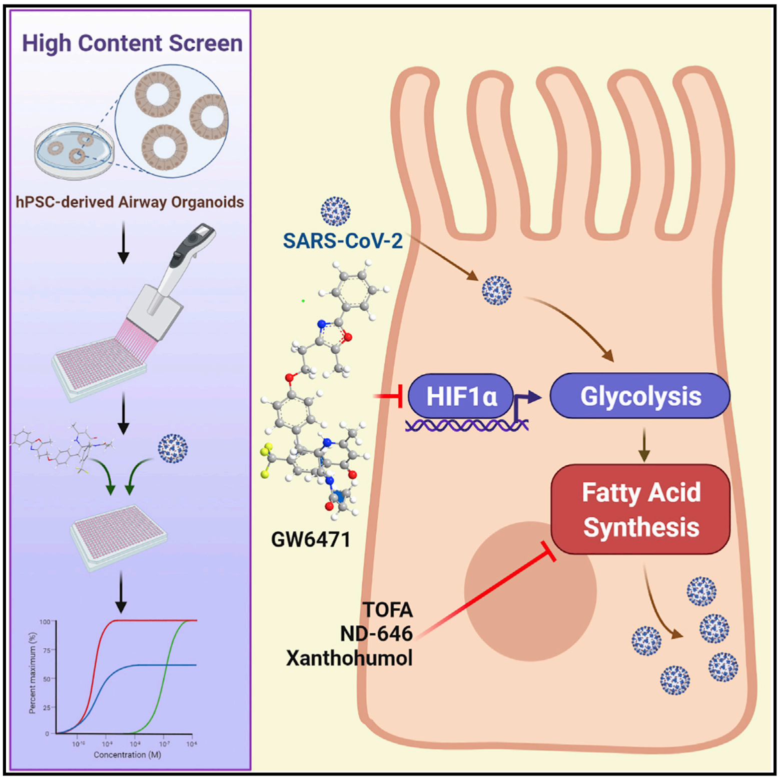

Authors

Xiaohua Duan, Xuming Tang, Manoj S. Nair, ..., David D. Ho, Todd Evans, Shuibing Chen

Correspondence

huiwang@shsmu.edu.cn (H.W.), res2025@med.cornell.edu (R.E.S.), dh2994@cumc.columbia.edu (D.D.H.), tre2003@med.cornell.edu (T.E.), shc2034@med.cornell.edu (S.C.)

In brief

Duan et al. develop an hPSC-derived airway organoid-based platform to monitor SARS-CoV-2 infection and screen for anti-viral drugs. From a high content chemical screen, they identify GW6471 that blocks SARS-CoV-2 infection in hPSC-derived airway organoids and hPSC-derived colonic organoids by inhibiting the HIF1α-glycolysis axis.

Highlights

- hPSC-derived airway organoids (hPSC-AOs) are permissive to SARS-CoV-2 infection
- An hPSC-AO-based high content screen identifies GW6471 blocking SARS-CoV-2 infection
- Chemical and genetic perturbation of HIF1α blocks SARS-CoV-2 infection
- Glycolysis is necessary for SARS-CoV-2 infection of human airway epithelium

Duan et al., 2021, Cell Reports *37*, 109920
November 9, 2021 © 2021 The Author(s).
https://doi.org/10.1016/j.celrep.2021.109920

CellPress

Cell Reports

CellPress OPEN ACCESS

Article

An airway organoid-based screen identifies a role for the HIF1α-glycolysis axis in SARS-CoV-2 infection

Xiaohua Duan,[1,5,8] Xuming Tang,[1,8] Manoj S. Nair,[2,8] Tuo Zhang,[3,8] Yunping Qiu,[4] Wei Zhang,[3] Pengfei Wang,[2] Yaoxing Huang,[2] Jenny Xiang,[3] Hui Wang,[5,*] Robert E. Schwartz,[6,7,*] David D. Ho,[2,*] Todd Evans,[1,*] and Shuibing Chen[1,9,*]

[1]Department of Surgery, Weill Cornell Medicine, 1300 York Ave., New York, NY 10065, USA
[2]Aaron Diamond AIDS Research Center, Columbia University Vagelos College of Physicians and Surgeons, New York, NY 10032, USA
[3]Genomics Resources Core Facility, Weill Cornell Medicine, New York, NY 10065, USA
[4]Department of Medicine, Fleischer Institute for Diabetes and Metabolism, Albert Einstein College of Medicine, Bronx, NY, USA
[5]State Key Laboratory of Oncogenes and Related Genes, Center for Single-Cell Omics, School of Public Health, Shanghai Jiao Tong University School of Medicine, Shanghai 200025, China
[6]Division of Gastroenterology and Hepatology, Department of Medicine, Weill Cornell Medicine, 1300 York Ave., New York, NY 10065, USA
[7]Department of Physiology, Biophysics and Systems Biology, Weill Cornell Medicine, 1300 York Ave., New York, NY 10065, USA
[8]These authors contributed equally
[9]Lead contact
*Correspondence: huiwang@shsmu.edu.cn (H.W.), res2025@med.cornell.edu (R.E.S.), dh2994@cumc.columbia.edu (D.D.H.), tre2003@med.cornell.edu (T.E.), shc2034@med.cornell.edu (S.C.)
https://doi.org/10.1016/j.celrep.2021.109920

SUMMARY

It is urgent to develop disease models to dissect mechanisms regulating severe acute respiratory syndrome coronavirus 2 (SARS-CoV-2) infection. Here, we derive airway organoids from human pluripotent stem cells (hPSC-AOs). The hPSC-AOs, particularly ciliated-like cells, are permissive to SARS-CoV-2 infection. Using this platform, we perform a high content screen and identify GW6471, which blocks SARS-CoV-2 infection. GW6471 can also block infection of the B.1.351 SARS-CoV-2 variant. RNA sequencing (RNA-seq) analysis suggests that GW6471 blocks SARS-CoV-2 infection at least in part by inhibiting hypoxia inducible factor 1 subunit alpha (HIF1α), which is further validated by chemical inhibitor and genetic perturbation targeting HIF1α. Metabolic profiling identifies decreased rates of glycolysis upon GW6471 treatment, consistent with transcriptome profiling. Finally, xanthohumol, 5-(tetradecyloxy)-2-furoic acid, and ND-646, three compounds that suppress fatty acid biosynthesis, also block SARS-CoV-2 infection. Together, a high content screen coupled with transcriptome and metabolic profiling reveals a key role of the HIF1α-glycolysis axis in mediating SARS-CoV-2 infection of human airway epithelium.

INTRODUCTION

Severe acute respiratory syndrome coronavirus 2 (SARS-CoV-2) is the cause of the coronavirus disease 2019 (COVID-19) pandemic, among the worst global health care crises of our time. There is a great and immediate need to develop new and relevant models to study virus biology and virus-host interactions and perform drug screens. Compared with typically used Vero cells (a kidney epithelial cell line derived from the African green monkey) or transformed carcinoma cell lines, human organoids may more faithfully recapitulate key aspects of viral infection and virus-host interactions. Several organoid models have been developed to study SARS-CoV-2 infection, including lung organoids (Han et al., 2021; Katsura et al., 2020; Lamers et al., 2021; Pei et al., 2021; Salahudeen et al., 2020; Samuel et al., 2020; Tiwari et al., 2021), bronchial organoids (Fang et al., 2021), intestinal organoids (Lamers et al., 2020; Zhou et al., 2020), colonic organoids (COs; Han et al., 2021), blood vessel and kidney organoids (Monteil et al., 2020), brain organoids (Jacob et al., 2020; Ramani et al., 2020; Zhang et al., 2020), and liver organoids (Yang et al., 2020; Zhao et al., 2020).

A recent study suggested that angiotensin converting enzyme 2 (ACE2), the putative receptor of SARS-CoV-2, is highly expressed in airway ciliated cells (Ziegler et al., 2020). Over the past several years, protocols have been reported to direct human pluripotent stem cell (hPSC) differentiation into various airway lineages (Chen et al., 2017, 2019b; Dye et al., 2015; Hawkins et al., 2017, 2021; Huang et al., 2014, 2015; Hurley et al., 2020; McCauley et al., 2017; Montoro et al., 2018; Mou et al., 2012). Here, we developed an hPSC-derived airway organoid (hPSC-AO) platform for monitoring infection of SARS-CoV-2 and to screen for inhibitors. Comparing with adult organoids, hPSC-AOs facilitate scaling, which is needed for high-throughput screening of small molecules and metabolic profiling. Using hPSC-AOs, we identified GW6471,

This is an open access article under the CC BY license (http://creativecommons.org/licenses/by/4.0/).

which blocks SARS-CoV-2 infection in the hPSC-AOs and hPSC-derived COs (hPSC-COs). Transcriptional and metabolic profiling reveals a key role for the HIF1α-glycolysis axis in mediating SARS-CoV-2 infection, which provides a target for anti-viral drug development.

RESULTS

Single-cell RNA-seq characterization of hPSC-AOs

We differentiated hPSCs to hPSC-AOs incorporating minor modifications into previously reported stepwise strategies (Hawkins et al., 2021; Huang et al., 2014; McCauley et al., 2017). Immunostaining confirmed that the hPSC-AOs contain MUC5AC$^+$ goblet cells and P63$^+$ basal cells (Figures S1A and S1B). To fully characterize the cellular complexity of the hPSC-AOs, single-cell transcriptome profiles identified *FOXJ1*$^+$ ciliated-like cells, *MUC5B*$^+$ goblet-like cells, *KRT5*$^+$*TP63*$^+$*KRT17*$^+$ basal cells, and *MKI67*$^+$*CDK1*$^+$*TOP2A*$^+$ proliferating cells (Figures 1A and 1B and S1C). The hPSC-derived ciliated-like cell population was enriched for adult human ciliated and proximal ciliated cell markers (Figure 1C). Correlation analysis of signature genes further validated identity of the hPSC-derived ciliated-like cell population in hPSC-AOs showing high similarity to adult human ciliated and proximal ciliated cells, but not alveolar epithelial type 1 or type 2 cells (Travaglini et al., 2020) (Figure 1D). Consistent with these data, the hPSC-derived ciliated-like population does not express markers for the alveolar epithelial type 2 cells, such as *SFTPD*, *SFTPC*, and *NAPSA* (Figure S1D). Both high-resolution phase contrast imaging (Figure 1E) and video (Video S1) validate the presence of multiple cilia in ciliated-like cells. The SARS-CoV-2 receptor, *ACE2* (Hoffmann et al., 2020), is highly expressed in the ciliated-like cell cluster (Figure 1F). *TMPRSS2* (Hoffmann et al., 2020) and *CTSL* (Ou et al., 2020), two key transmembrane proteases used for SARS-CoV-2 entry; *FURIN*, a pro-protein convertase that pre-activates SARS-CoV-2 (Shang et al., 2020); and *NRP1*, a host factor of SARS-CoV-2 infection (Cantuti-Castelvetri et al., 2020; Daly et al., 2020), are expressed more widely, but also enriched in the ciliated-like cell cluster (Figure 1F). Immunostaining analysis further validated that ACE2 is expressed in acetyl-α-tubulin (AATB)$^+$FOXJ1$^+$ ciliated-like cells (Figures 1G and 1H).

hPSC-AOs are permissive to SARS-CoV-2 infection

To determine their permissiveness, hPSC-AOs were infected with SARS-CoV-2 (USA-WA1/2020, MOI = 0.2). At 48 h post-infection (hpi), qRT-PCR analysis using primers targeting subgenomic N transcripts confirmed that significant viral replication could be detected at the RNA level in infected hPSC-AOs (Figure 2A). Immunostaining for SARS-CoV-2 nucleocapsid (SARS-N) protein (Figure 2B) confirmed robust SARS-CoV-2 infection of hPSC-AOs. The 3D confocal imaging further validated the presence of SARS-N in ciliated-like cells (Figure 2C; Video S2). In addition, the transcriptome profiling (Figure 2D) confirmed robust SARS-CoV-2 infection of hPSC-AOs. Moreover, plotting these RNA-sequencing (RNA-seq) datasets by principle-component analysis (PCA; Figure 2E) and clustering analysis (Figure 2F) demonstrated that the infected hPSC-AOs occupy a distinct transcriptional space compared with mock-infected hPSC-AOs. A volcano plot of SARS-CoV-2-infected hPSC-AOs compared with mock-treated organoids revealed the upregulation of SARS-CoV-2-associated transcripts and the robust induction of chemokines, including *CXCL1*, *CXCL2*, *CXCL3*, *CXCL10*, and *CXCL11* (Figure 2G), as has previously been observed in SARS-CoV-2-infected lung tissues (Blanco-Melo et al., 2020; Han et al., 2021; Yang et al., 2020). Interestingly, interferon-pathway-associated genes, such as *IFIT1*, *IFIT2*, *IFIT3*, *IFI44L*, *IFNB1*, *OAS2*, and *EGR1*, are also upregulated in SARS-CoV-2-infected hPSC-AOs (Figure 2G).

A high content chemical screen using hPSC-AOs identifies GW6471 blocking SARS-CoV-2 infection

To identify small molecules capable of blocking SARS-CoV-2 virus infection, hPSC-AOs were deposited onto 384-well plates followed by addition of chemicals from a collection of agonists and antagonists of known signaling pathways and US Food and Drug Administration (FDA)-approved drugs. The compound information is shown in Table S1. Two hours post-treatment, organoids were infected with SARS-CoV-2 virus at MOI = 0.2. At 48 hpi, the organoids were fixed and analyzed for the percentage of SARS-N$^+$ cells. The wells which the Z score was less than -2 were chosen as primary hit compounds (Figure 3A). Hit compounds were evaluated for efficacy and cytotoxicity at different concentrations (Figures 3B and S2A). One compound, GW6471, was confirmed to decrease the percentage of SARS-N$^+$ cells through a dose-dependent manner, independent of cytotoxicity (half maximal effective concentration [EC_{50}] = 2.1 μM; Figures 3B and 3C and S2B). qRT-PCR analysis showed a significant reduction of replicating virus in GW6471-treated hPSC-AOs (Figure 3D). Immunostaining confirmed a significant reduction of SARS-N$^+$ cells detected among FOXJ1$^+$ ciliated-like cells in hPSC-AOs treated with 10 μM GW6471 (Figures 3E and 3F). The compound was also evaluated for anti-SARS-CoV-2 activity post-infection. hPSC-AOs were infected with SARS-CoV-2 (MOI = 0.2). After 24 hpi, these hPSC-AOs were treated with 10 μM GW6471. At 48 hpi, both viral RNA (Figure 3G) and SARS-N$^+$ cells (Figures 3H and 3I) were significantly decreased in the compound-treated hPSC-AOs. Finally, GW6471 was evaluated for its capacity to block infection of the B.1.351 SARS-CoV-2 variant. Both viral RNA (Figure 3J) and SARS-N$^+$ cells (Figures 3K and 3L) were significantly decreased in the GW6471-treated hPSC-AOs.

Previously, we showed that hPSC-COs are permissive to SARS-CoV-2 infection (Han et al., 2021). We further validated GW6471 as an anti-SARS-CoV-2 compound using hPSC-COs. The hPSC-COs were pretreated with GW6471 prior to infection with SARS-CoV-2. A reduction of subgenomic viral RNA (Figure 3M) and a decrease of SARS-N$^+$ cells were found in GW6471-treated hPSC-COs, which was similar to our observations with hPSC-AOs. (Figures 3N and 3O). To determine whether GW6471 has anti-viral activity post-infection, hPSC-COs were infected with SARS-CoV-2 (MOI = 3). At 24 hpi, hPSC-COs were treated with 10 μM GW6471. At 48 hpi, both viral RNA (Figure 3P) and the number of SARS-N$^+$ cells (Figures 3Q and 3R) were significantly decreased in the GW6471-treated hPSC-COs.

To determine whether GW6471 affects viral particle stability, a concentrated SARS-CoV-2 virus preparation was pre-incubated

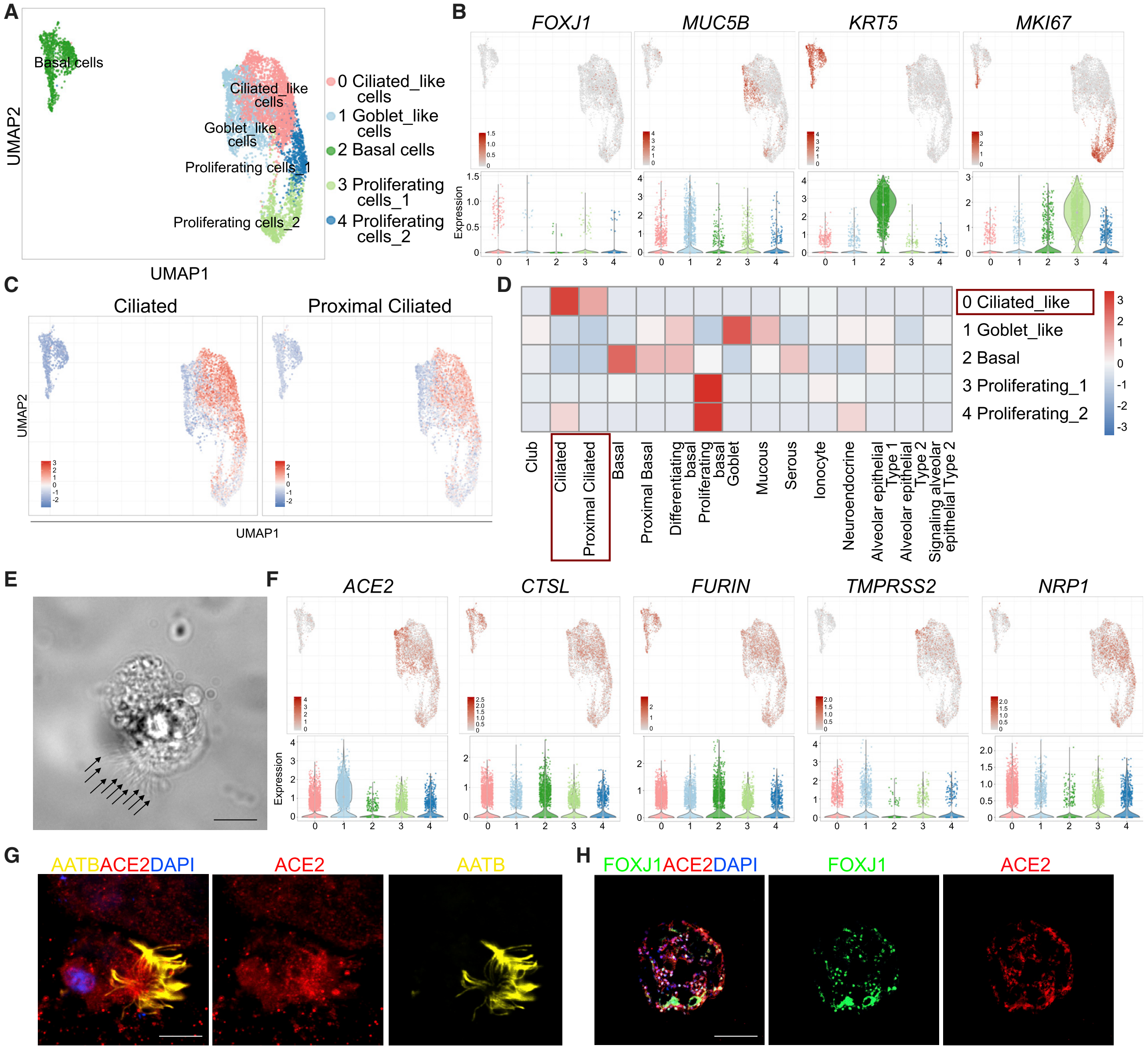

Figure 1. Single-cell RNA-seq analysis of hPSC-AOs

(A) Uniform manifold approximation and projection (UMAP) plot illustrating five cell clusters in the hPSC-AOs. n = 1 biological replicate.

(B) UMAP and violin plots showing the expression of genes *FOXJ1*, *MUC5B*, *KRT5*, and *MKI67*.

(C) Enrichment analysis of hPSC-AOs using genes highly expressed in adult human ciliated or proximal ciliated cells.

(D) Correlation analysis of genes with cell fates in hPSC-AOs and adult human lung cells.

(E) Phase contrast image of a representative ciliated-like cell. Scale bar, 30 μm.

(F) UMAP and violin plots showing the expression of SARS-CoV-2 entry factors, including *ACE2*, *CTSL*, *FURIN*, *TMPRSS2*, and *NRP1*.

(G) Representative confocal images of hPSC-AOs co-stained with antibodies recognizing ACE2 and cilia marker acetyl-α-tubulin (AATB). 4′,6-diamidino-2-phenylindole (DAPI) stains nuclei. Scale bar, 20 μm.

(H) Representative confocal images of hPSC-AOs co-stained with antibodies recognizing ACE2 and ciliated cell marker FOXJ1. DAPI stains nuclei. Scale bar, 100 μm.

See also Figure S1 and Video S1.

with 10 μM GW6471 for 3 h. At 48 hpi, no significant difference was detected for the percentage of SARS-N^{+} cells comparing hPSC-AOs infected with SARS-CoV-2 that was pre-incubated with GW6471 or control (Figures S3A and S3B), suggesting that GW6471 does not affect the stability of viral particles. To determine the impact of GW6471 on viral entry, we used a luciferase-based pseudovirus as reported in our previous publication (Han et al., 2021). GW6471 does not block viral entry

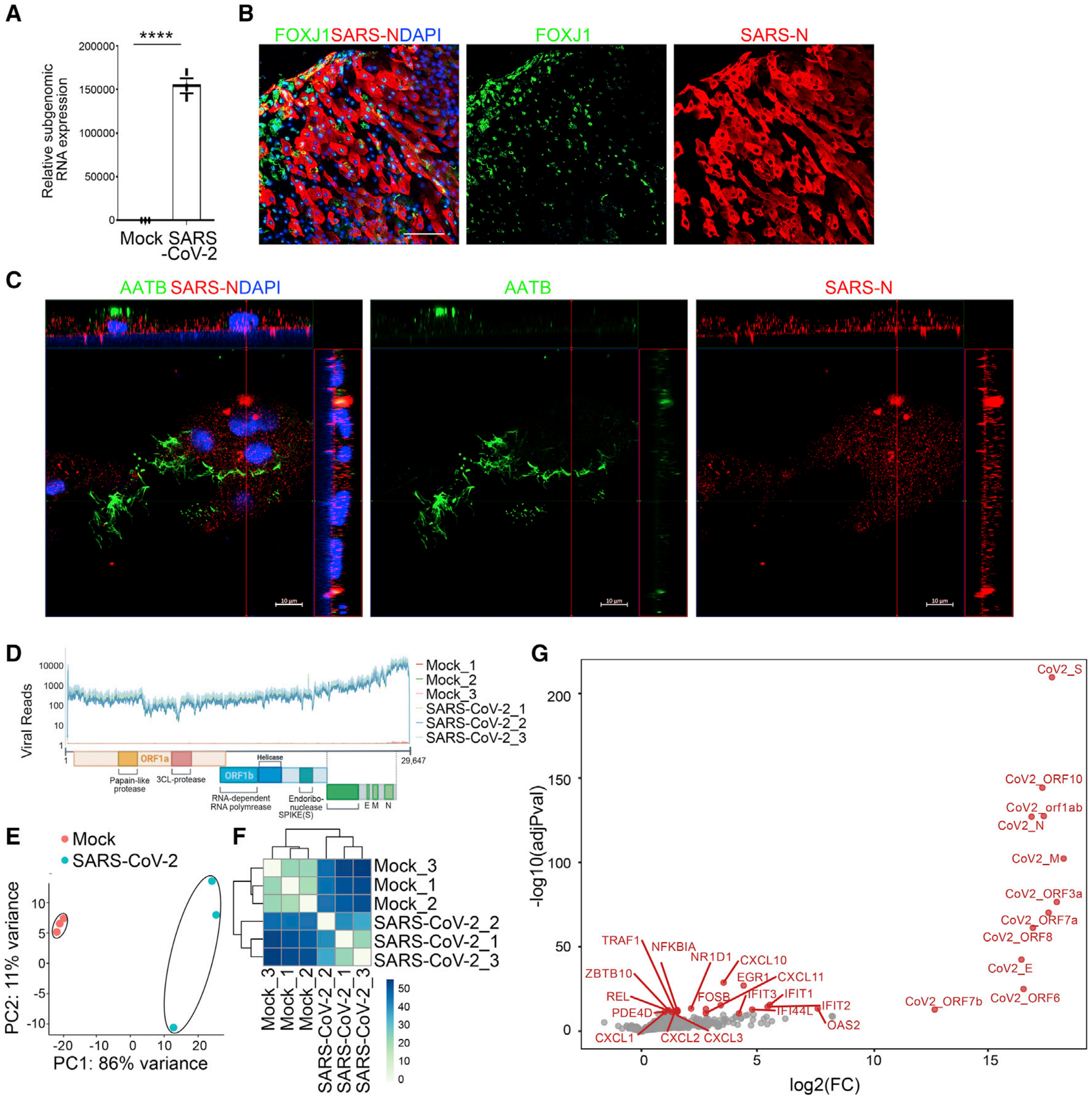

Figure 2. hPSC-AOs are permissive to SARS-CoV-2 infection

(A) Relative SARS-CoV-2 RNA expression levels in hPSC-AOs at 48 hpi (MOI = 0.2). Total viral RNA from infected hPSC-AOs was analyzed by qRT-PCR for the presence of N transcripts relative to *ACTB*.

(B) Representative confocal images of hPSC-AOs at 48 hpi (MOI = 0.2) co-stained with antibodies recognizing SARS-CoV-2 nucleocapsid (SARS-N) protein and ciliated cell marker FOXJ1. Scale bar, 100 μm.

(C) Representative 3D confocal images of hPSC-AOs at 48 hpi (MOI = 0.2) co-stained with antibodies recognizing SARS-N and AATB. Scale bar, 10 μm.

(D) Read coverage on viral transcriptome in the mock- and SARS-CoV-2-infected hPSC-AOs (MOI = 0.2). Schematic shows the SARS-CoV-2 genome. Coverage is normalized per million reads.

(E and F) PCA (E) and sample clustering (F) on the mock- and SARS-CoV-2-infected hPSC-AOs.

(G) Volcano plot showing the gene expression changes between mock- and SARS-CoV-2-infected hPSC-AOs.

Data in (A) are presented as mean ± SEM (n = 3 biological replicates). The p values were calculated by unpaired two-tailed Student's t test. ****$p < 0.0001$.

See also Video S2.

(Figure S3C). Consistent with this observation, ACE2 expression levels are not changed after GW6471 treatment (Figure S3D).

The HIF1α pathway and SARS-CoV-2 infection

To examine the mechanism of action for GW6471 in the context of our organoid-based SARS-CoV-2 infection model, RNA-seq analysis was carried out to compare the transcriptome profiles of control and GW6471-treated hPSC-AOs or hPSC-COs. PCA plots (Figure 4A) and clustering analysis (Figure 4B) showed that profiles of control and GW6471-treated hPSC-AOs clustered separately. Volcano plot (Figure 4C) and Ingenuity pathway analysis (IPA; Figure 4D; Table S2) highlighted downregulation of the HIF1α

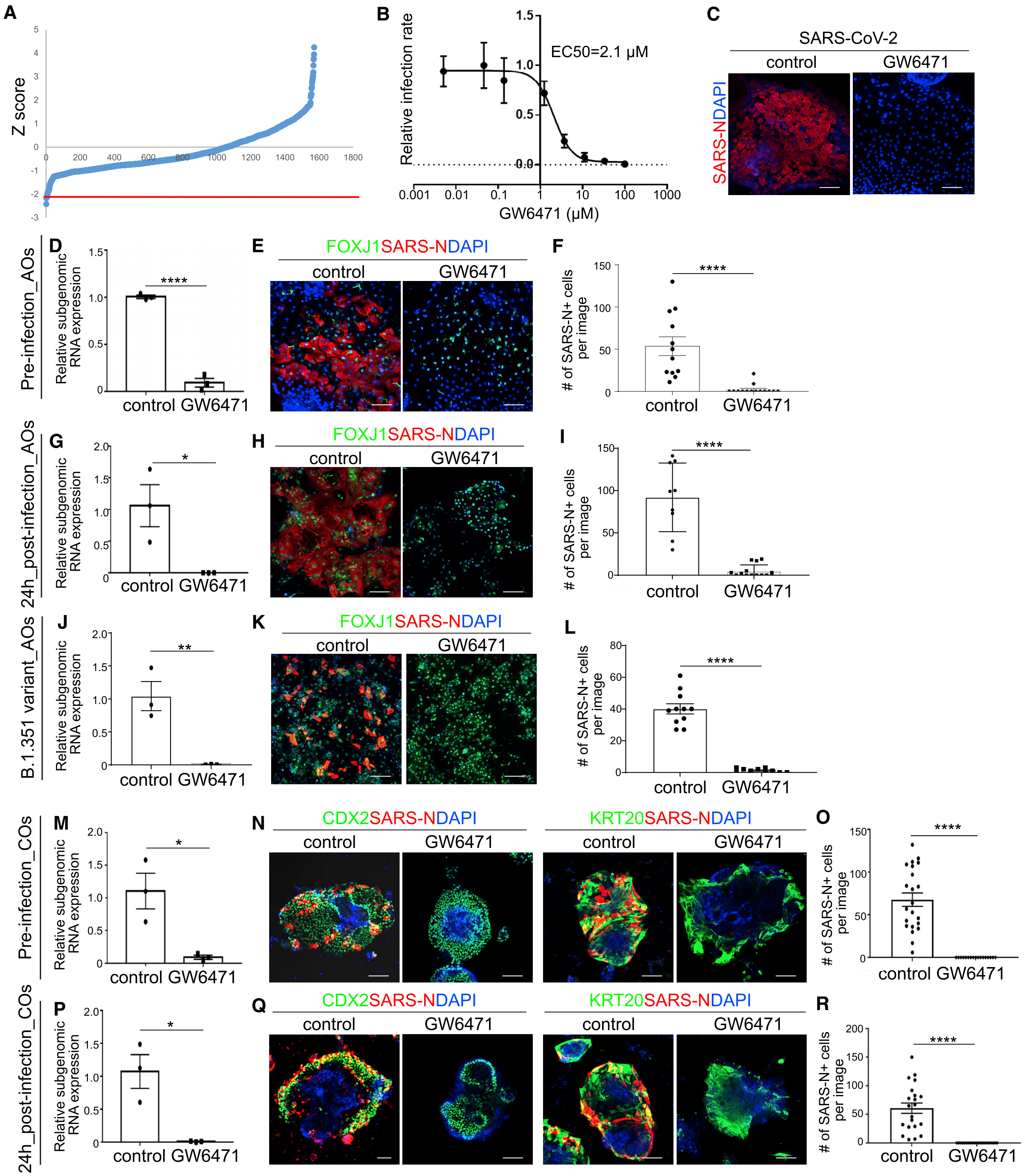

Figure 3. An hPSC-AO-based high-throughput chemical screen identifies GW6471 that blocks SARS-CoV-2 infection

(A) Primary screening results. The x axis is the compound number. The y axis is the *Z* score. Red line indicates *Z* score = −2.

(B) Efficacy curve of GW6471. Data are presented as mean ± SD. n = 3 biological replicates.

(C) Representative confocal images of control or GW6471-treated hPSC-AOs at 48 hpi (MOI = 0.2). Scale bar, 100 μm.

(D) qRT-PCR analysis for viral N subgenomic RNA in hPSC-AOs, which were pretreated with control or 10 μM GW6471 at 48 hpi (MOI = 0.2). Data are presented as mean ± SEM. n = 3 biological replicates.

(legend continued on next page)

signaling pathway in treated cells. Likewise, RNA-seq data from control and GW6471-treated hPSC-COs had profiles that clustered separately, as indicated by PCA plots (Figure 4E) and clustering analysis (Figure 4F). Volcano plot (Figure 4G) and IPA (Figure 4H; Table S2) also highlighted downregulation of the HIF1α signaling pathway. Although several cell-cycle-associated pathways were identified in GW6471-treated hPSC-AOs, GW6471 treatment does not affect cell proliferation (Figures S3E–S3H). In the absence of SARS-CoV-2 infection, GW6471 does not affect chemokine expression (Figure S3I) or the interferon pathway (Figure S3J). In the presence of SARS-CoV-2 infection, GW6471 treatment showed a trend to decrease the expression of chemokines (Figure S3K), but no consistent trend for impacting genes involved in interferon signaling (Figure S3L).

To test whether decreased HIF1α signaling is functionally relevant to controlling infection, the compound chetomin, a known small molecule suppressor for transcriptional activation of the HIF1α pathway, was tested (Kung et al., 2004). Both viral RNA replication (Figure 4I) and the number of SARS-N$^+$ cells (Figures 4J and 4K) were significantly decreased following SARS-CoV-2 infection of hPSC-AOs, when treated with 1 μM chetomin. Similar to GW6471, chetomin does not affect chemokine expression (Figure S4A) or the interferon pathway (Figure S4B) in the absence of SARS-CoV-2. In the presence of SARS-CoV-2 infection, chetomin treatment showed a trend to decrease chemokine expression levels (Figure S4C), but no consistent trend for impacting genes involved in interferon signaling (Figure S4D). We used short hairpin RNAs (shRNAs) to knockdown expression levels of HIF1α (Table S3). qRT-PCR analysis confirmed the knockdown efficiency and specificity (Figure S4C). hPSC-AOs carrying scrambled shRNA or shHIF1α were infected with SARS-CoV-2 (MOI = 0.2). At 48 hpi, both qRT-PCR and immunostaining data showed decreased viral infection in hPSC-AOs expressing shHIF1α (Figures 4L–4N).

Metabolic profiling identifies the role of glycolysis in SARS-CoV-2 infection

Metabolic profiling was performed to compare mock- versus SARS-CoV-2-infected hPSC-AOs and (following SARS-CoV-2 infection) control versus GW6471-treated hPSC-AOs. Profiles from SARS-CoV-2 and mock-infected hPSC-AOs clustered separately (Figure 5A). Fatty acids, such as oleic acid, palmitic acid, palmitoleic acid, and stearic acid, and amino acids, such as glycine, isoleucine, threonine, and serine, were all increased in SARS-CoV-2-infected hPSC-AOs (Figure 5B). In addition, citric acid and D-glucose-6-phosphate levels also increased in SARS-CoV-2-infected hPSC-AOs (Figure 5B). Profiles from hPSC-AOs that were GW6471 or control-treated following SARS-CoV-2 infection clustered separately (Figure 5C). Compared with controls, GW6471-treated hPSC-AOs showed decreased levels of fatty acids, including oleic acid, palmitic acid, palmitoleic acid, and amino acids, as well as D-glucose-6-phosphate and citric acid (Figure 5D). Transcriptome profiling confirmed that genes encoding enzymes in the glycolysis pathway are suppressed in GW6471-treated conditions (Figure 5E). This is consistent with downregulation of the glycolysis pathway in GW6471-treated hPSC-COs (Figure 4H).

HIF1α signaling controls glycolysis upon SARS-CoV-2 infection

To determine whether HIF1α signaling controls glycolysis in the hPSC-AOs, we compared transcript profiles of the control and chetomin-treated samples. PCA plots (Figure 6A) and clustering analysis (Figure 6B) showed that profiles of control and chetomin-treated hPSC-AOs clustered separately. Consistent with the results of GW6471-treated conditions (Figure 5E), genes encoding enzymes in the glycolysis pathway were suppressed in chetomin-treated conditions (Figure 6C). RNA-seq was also applied to analyze the hPSC-AOs expressing shHIF1α upon SARS-CoV-2 infection. Profiles from hPSC-AOs expressing scrambled RNA or shHIF1α clustered separately (Figures 6D and 6E). RNA-seq analysis confirmed downregulation of the HIF1α pathway in hPSC-AOs expressing shHIF1α (Figure 6F). Interestingly, the coronavirus replication pathway was also found to be downregulated in hPSC-AOs expressing shHIF1α upon SARS-CoV-2 infection (Figure 6F). Genes involved in the glycolysis pathway are also downregulated in hPSC-AOs expressing shHIF1α (Figure 6G; Table S4). The hPSC-AOs expressing shHIF1α show slightly

(E and F) Representative confocal images (E) and quantification (F) of SARS-N in hPSC-AOs, which were pretreated with control or 10 μM GW6471 at 48 hpi (MOI = 0.2). Scale bar, 100 μm. Data are presented as mean ± SEM. n = 6 biological replicates.
(G) Relative SARS-CoV-2 viral RNA expression levels at 48 hpi in hPSC-AOs infected with SARS-CoV-2 virus (MOI = 0.2) and 24 h later exposed to control or 10 μM GW6471 treatment. Data are presented as mean ± SEM. n = 3 biological replicates.
(H and I) Representative confocal images (H) and quantification (I) of SARS-N at 48 hpi of hPSC-AOs infected with SARS-CoV-2 virus (MOI = 0.2) and 24 h later exposed to control or 10 μM GW6471 treatment. Scale bar, 100 μm. Data are presented as mean ± SEM. n = 6 biological replicates.
(J) qRT-PCR analysis for viral N single-guide RNA (sgRNA) at 48 hpi of hPSC-AOs, which were pretreated with control or 10 μM GW6471 (variant B.1.351, MOI = 0.2). Data are presented as mean ± SEM. n = 3 biological replicates.
(K and L) Representative confocal images (K) and quantification (L) of SARS-N at 48 hpi of hPSC-AOs, which were pretreated with control or 10 μM GW6471 (variant B.1.351, MOI = 0.2). Scale bar, 100 μm. Data are presented as mean ± SEM. n = 6 biological replicates.
(M) qRT-PCR analysis for viral N sgRNA at 48 hpi of hPSC-COs, which were pretreated with control or 10 μM GW6471 (MOI = 3). Data are presented as mean ± SEM. n = 3 biological replicates.
(N and O) Representative confocal images (N) and quantification (O) at 48 hpi of SARS-N of hPSC-COs, which were pretreated with control or 10 μM GW6471 (MOI = 3). Scale bar, 100 μm. Data are presented as mean ± SEM. n = 6 biological replicates.
(P) Relative SARS-CoV-2 viral RNA expression levels at 48 hpi of hPSC-AOs infected with SARS-CoV-2 virus (MOI = 3) and 24 h later followed by control or 10 μM GW6471 treatment. Data are presented as mean ± SEM. n = 3 biological replicates.
(Q and R) Representative confocal images (Q) and quantification (R) of SARS-N at 48 hpi of hPSC-AOs infected with SARS-CoV-2 virus (MOI = 3) and 24 h later followed by control or 10 μM GW6471 treatment. Scale bar, 100 μm. Data are presented as mean ± SEM. n = 6 biological replicates.
The p values were calculated by unpaired two-tailed Student's t test. *p < 0.05 and ***p < 0.0001.
See also Figures S2 and S3 and Table S1.

CellPress
OPEN ACCESS

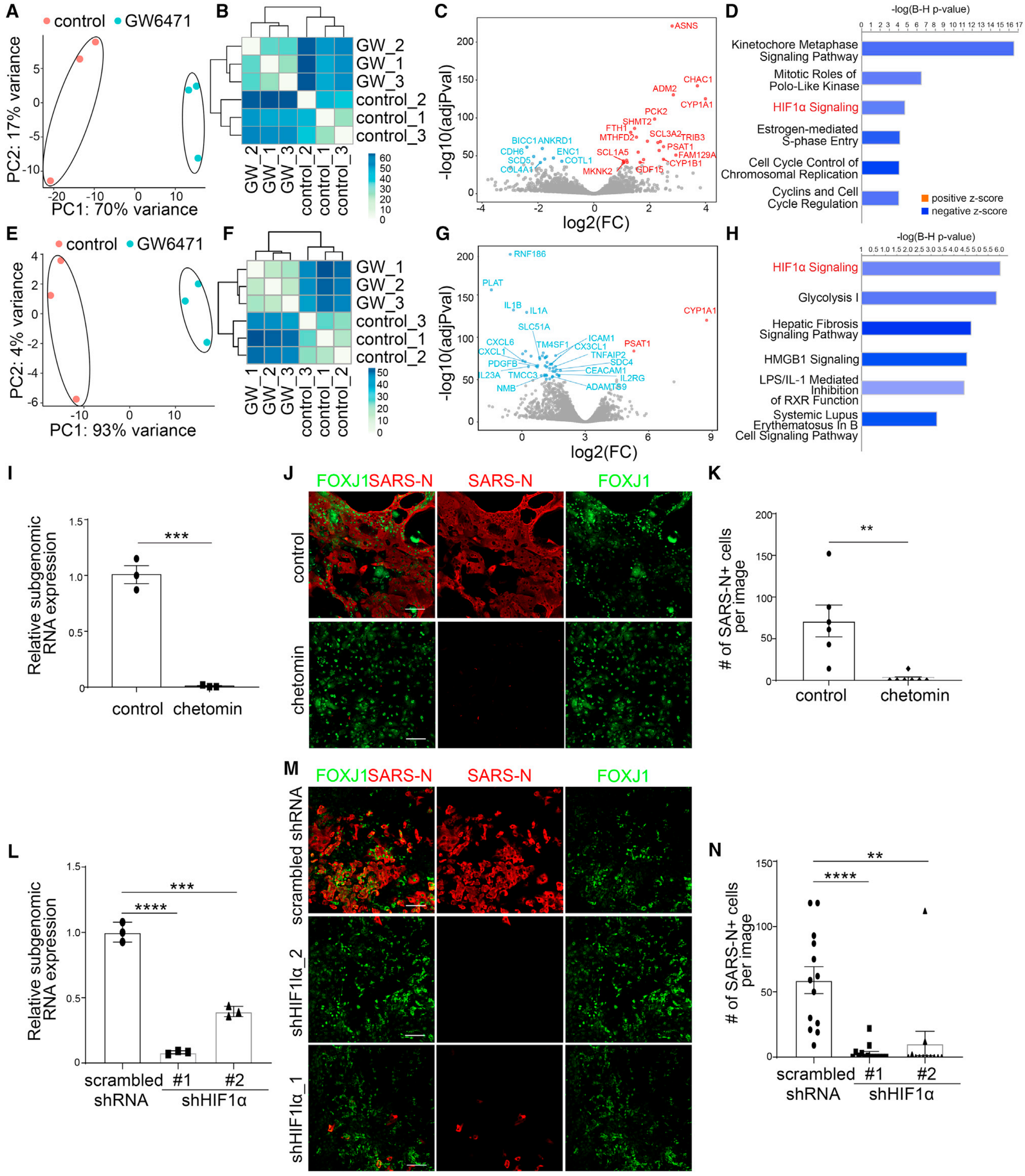

Figure 4. GW6471 blocks SARS-CoV-2 infection by inhibiting the HIF1α pathway

(A and B) PCA (A) and sample clustering (B) on the control- and 10 μM GW6471-treated hPSC-AOs at 48 hpi (MOI = 0.2).

(C) Volcano plot showing the expression changes between control- and 10 μM GW6471-treated hPSC-AOs at 48 hpi (MOI = 0.2).

(D) Enriched pathways in GW6471- versus control-treated hPSC-AOs at 48 hpi.

(E and F) PCA (E) and sample clustering (F) on the control- and 10 μM GW6471-treated hPSC-COs at 48 hpi (MOI = 1).

(G) Volcano plot showing the expression changes between control- and 10 μM GW6471-treated hPSC-COs at 48 hpi (MOI = 1).

(legend continued on next page)

decreased levels of ACE2 expression, but this difference seems unlikely to be sufficient to explain the significant decrease of SARS-CoV-2 infection in hPSC-AOs expressing shHIF1α (Figure S4F). Metabolic profiling also confirmed the decreased levels of fatty acids, including palmitic acid, oleic acid, and palmitoleic acid, as well as cholesterol, in hPSC-AOs expressing shHIF1α (Figures 6H and 6I). SARS-CoV-2-infected hPSC-AOs were next treated with xanthohumol, a prenylated flavonoid that suppresses fatty acid and cholesterol biosynthesis (Miyata et al., 2015) and the glycolysis pathway (Yuan et al., 2020). Both viral RNA levels (Figure 6J) and the number of SARS-N$^+$ cells (Figures 6K and 6L) were significantly decreased in the 10 μM xanthohumol-treated hPSC-AOs. Finally, 5-(tetradecyloxy)-2-furoic acid (TOFA) (Parker et al., 1977) and ND-646 (Svensson et al., 2016), two inhibitors of acetyl-coenzyme A (CoA) carboxylase 1, the key enzyme involved in fatty acid *de novo* synthesis, were tested to further validate the role of fatty acid metabolism in SARS-CoV-2 infection. Both qRT-PCR (Figure 6M) and immunostaining (Figures 6N and 6O) confirmed that TOFA or ND-646 blocks SARS-CoV-2 infection in hPSC-AOs.

DISCUSSION

Morbidity and mortality from COVID-19 are largely attributed to the acute viral pneumonitis that evolves to acute respiratory distress syndrome. ACE2 is highly expressed in multiciliated cells of the human airway (Ziegler et al., 2020). We successfully developed an hPSC-AO platform, containing AATB$^+$FOXJ1$^+$ ciliated-like cells that express ACE2. RNA-seq analysis of infected organoids revealed upregulation of cytokine/chemokine signaling, which mimics the inflammatory changes observed in primary human COVID-19 pulmonary infections (Blanco-Melo et al., 2020). We adapted the hPSC-AOs to a high content platform to screen for chemicals that block SARS-CoV-2 infection. From a pilot screen, we identified GW6471 that blocks SARS-CoV-2 infection not only in hPSC-AOs but also in hPSC-COs, indicating that the anti-SARS-CoV-2 activity of GW6471 functions across different organ systems. Transcriptome profiles suggested that GW6471 blocks SARS-CoV-2 infection at least in part by inhibiting HIF1α, which was further validated using a known HIF1α inhibitor, chetomin. At a molecular level, chetomin disrupts the structure of the cysteine/histidine-rich 1 (CH1) domain of p300 and precludes its interaction with HIF, thereby attenuating hypoxia-inducible transcription (Kung et al., 2004). We further validated the role of HIF1α in SARS-CoV-2 infection using shRNAs. HIF1α has been shown to modulate virus replication in primary airway epithelial cells infected with respiratory syncytial virus (Morris et al., 2020). In addition, studies using monocytes showed that SARS-CoV-2 infection triggers mitochondrial reactive oxygen species production, which induced HIF1α stabilization and glycolysis. In our study, we found that GW6471 downregulates the HIF1 pathway in both hPSC-AOs (Figures 4A–4D) and hPSC-COs upon SARS-CoV-2 infection (Figures 4E–4H) and that knockdown of HIF1α (Figures 4I–4K) or use of a specific HIF1α inhibitor (Figures 4L and 4M) blocks SARS-CoV-2 infection. Bojkova et al. (2020) reported changes in the HIF1 pathway and carbon metabolism in SARS-CoV-2-infected Caco-2 cells. They further showed that blocking glycolysis with 2-deoxy-D-glucose, an inhibitor of hexokinase (the rate-limiting enzyme in glycolysis), prevented SARS-CoV-2 replication in Caco-2 cells (Bojkova et al., 2020). A recent proteo-transcriptomics analysis in SARS-CoV-2-infected Huh7 cells found a dose-dependent increase of serine/threonine kinase-mechanistic target of rapamycin kinase (Akt-mTOR) signaling, but surprisingly, a decrease in activity of HIF1α. However, while 68% of differentially expressed HIF1 pathway genes were down-regulated, the other 32% were upregulated (Appelberg et al., 2020). In addition, hypoxemia is the typical characteristic in patients with severe COVID-19, which is consistent with our findings (Berenguer et al., 2020; Petrilli et al., 2020). Together, the studies suggest that the HIF1 pathway might play a cell type and context dependent role to support SARS-CoV-2 infection.

- Metabolic profiling suggested decreased levels of fatty acids upon GW6471 treatment (Figure 5D) or knockdown of HIF1α (Figure 6I). GW6471 was previously reported as a PPARα antagonist (Xu et al., 2002). Saturated and unsaturated fatty acids are endogenous ligands for peroxisome proliferator activated receptor alpha (PPARα). Polyunsaturated fatty acids, ω6-polyunsaturated fatty acids, and saturated fatty acids have been shown to directly bind to PPARα. In addition, PPARα has been shown to play a critical role in hepatic fatty acid oxidation and ketogenesis based on analysis of *PPARα*$^{-/-}$ mice (Kersten, 2014). Here, we found that GW6471-treated hPSC-AOs showed decreased levels of fatty acids compared with control-treated hPSC-AOs (Figure 5D), suggesting that there might be a cell-type-dependent feedback loop that controls fatty acid levels in hPSC-AOs. In hPSC-AOs, GW6471 inhibits the HIF1α-glycolysis axis. The pyruvate produced by glycolysis is an important intermediary in the conversion of carbohydrates into fatty acids and cholesterol, which might partially explain the decrease of fatty acids in GW6471-treated hPSC-AOs. Many viruses, including

(H) Enriched pathways in GW6471- versus control-treated hPSC-COs at 48 hpi.
(I) qRT-PCR analysis for viral N sgRNA of hPSC-AOs treated with control or 1 μM chetomin at 48 hpi (MOI = 0.2). Data are presented as mean ± SEM. n = 3 biological replicates.
(J and K) Representative confocal images (J) and quantification (K) of SARS-N$^+$ cells of hPSC-AOs, which were treated with control or 1 μM chetomin at 48 hpi (MOI = 0.2). Scale bar, 100 μm. Data are presented as mean ± SEM. n = 6 biological replicates.
(L) qRT-PCR analysis for viral N sgRNA of hPSC-AOs expressing shHIF1α or scrambled shRNA at 48 hpi (MOI = 0.2). Data was presented as mean ± SEM. n = 3 biological replicates.
(M and N) Representative confocal images (M) and quantification (N) of SARS-N$^+$ cells of hPSC-AOs expressing shHIF1α or scrambled shRNA at 48 hpi (MOI = 0.2). Scale bar, 100 μm. Data are presented as mean ± SEM. n = 6 biological replicates.
The p values were calculated by unpaired two-tailed Student's t test. ***$p < 0.001$ and ****$p < 0.001$.
See also Figures S3 and S4 and Tables S2 and S3.

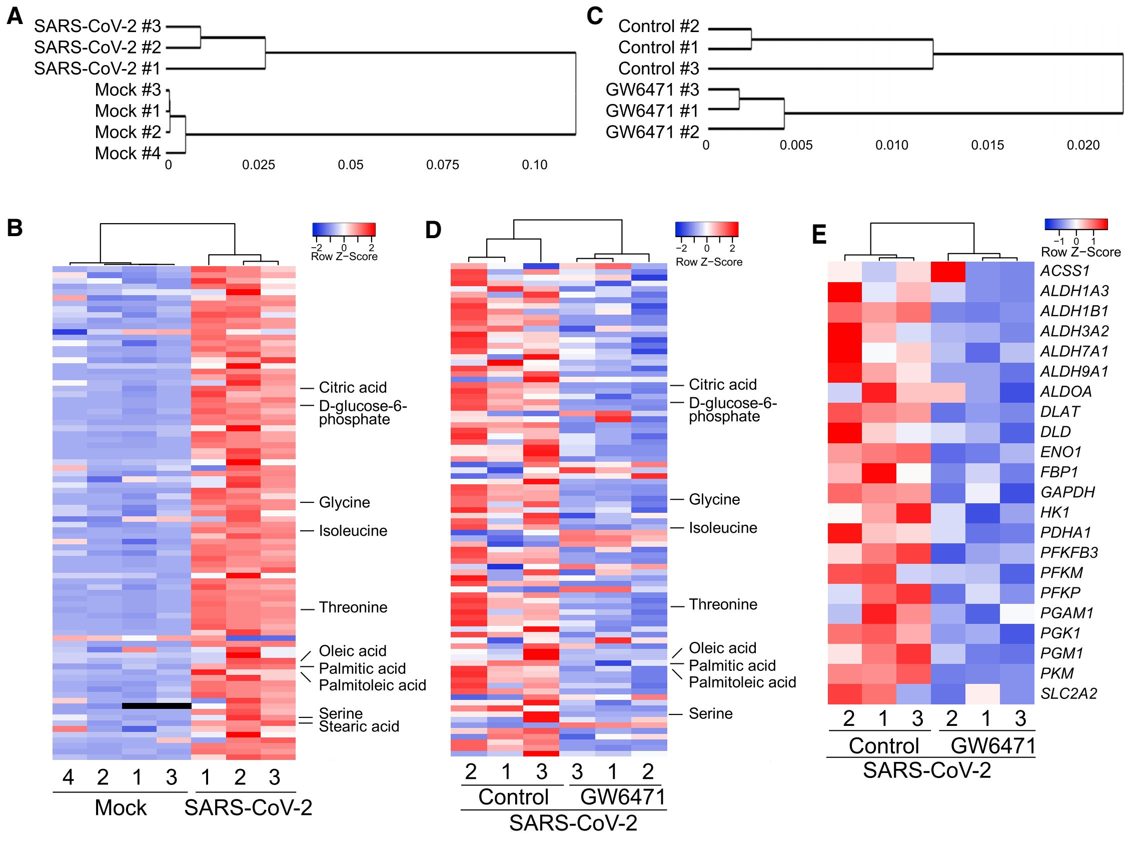

Figure 5. Metabolic profiling identifies a key role for glycolysis in SARS-CoV-2 infection

(A and B) Hierarchical clustering analysis (A) and heatmap (B) of metabolic profiles for mock- or SARS-CoV-2 (MOI = 0.2)-infected hPSC-AOs at 48 hpi. n = 4 biological replicates for mock and N = 3 biological replicates for SARS-CoV-2.

(C and D) Hierarchical clustering analysis (C) and heatmap (D) of metabolic profiles for control- or 10 μM GW6471-treated hPSC-AOs at 48 hpi (MOI = 0.2). n = 3 biological replicates.

(E) Heatmap of genes encoding enzymes involved in the glycolytic pathway of control- or 10 μM GW6471-treated hPSC-AOs at 48 hpi (MOI = 0.2). n = 3 biological replicates.

cowpea mosaic virus (Carette et al., 2000), poliovirus (Cherry et al., 2006; Guinea and Carrasco, 1990), tombusvirus (Sharma et al., 2010, 2011), and Semliki Forest virus (Perez et al., 1991), require fatty acids for their replication and assembly. Modulation of fatty acid metabolism has been shown to impact the replication of several viruses, including hepatitis C virus (Nasheri et al., 2013), and Old World alphaviruses (Bakhache et al., 2019). In addition, a preprint shows that orlistat, a drug that inhibits lipases and also fatty acid synthase, and triacsin C, which inhibits long chain acyl-CoA synthetases, block SARS-CoV-2 replication in Vero cells and Calu-3 cells (Silvas et al., 2020). Consistent with these observations, RNA-seq analysis identified downregulation of the coronavirus replication pathway in hPSC-AOs expressing shRNA against HIF1α (Figure 6F). Since fatty acids are also involved in viral particle formation (Herker et al., 2010) and other steps of viral cycle as discussed in several reviews (Chukkapalli et al., 2012; Heaton and Randall, 2011; Lorizate and Kräusslich, 2011), GW6471 might also affect multiple steps of the viral life cycle.

- Finally, we performed metabolic profiling using SARS-CoV-2-infected hPSC-AOs and found increased levels of D-glucose-6 phosphate, citric acid, fatty acids, and amino acids, suggesting that infection stimulates glycolysis as part of a metabolic rewiring. Indeed, GW6471 treatment and knockdown of HIF1α, which block SARS-CoV-2 infection, lead to decreased levels of D-glucose-6 phosphate, citric acid, fatty acids, and amino acids. The data are consistent with the metabolic state being a key regulator of SARS-CoV-2 infection. The metabolic analysis of the sera of patients with COVID-19 identified increased circulating levels of free fatty acids (Appelberg et al., 2020), which is consistent with our data using hPSC-AOs. HIF1α is known to regulate glycolysis in immune cells, including monocytes (Appelberg et al., 2020), macrophages (Chen et al., 2019a), and dendritic cells (Liu et al., 2019). Consistent with these observations, glycolysis-associated genes are upregulated in SARS-CoV-2-infected hPSC-lung organoids, pancreatic endocrine cells, and lung autopsy samples from patients with COVID-19 (Figures S4G–S4I). Our data are consistent with a recent study showing that elevated glucose levels

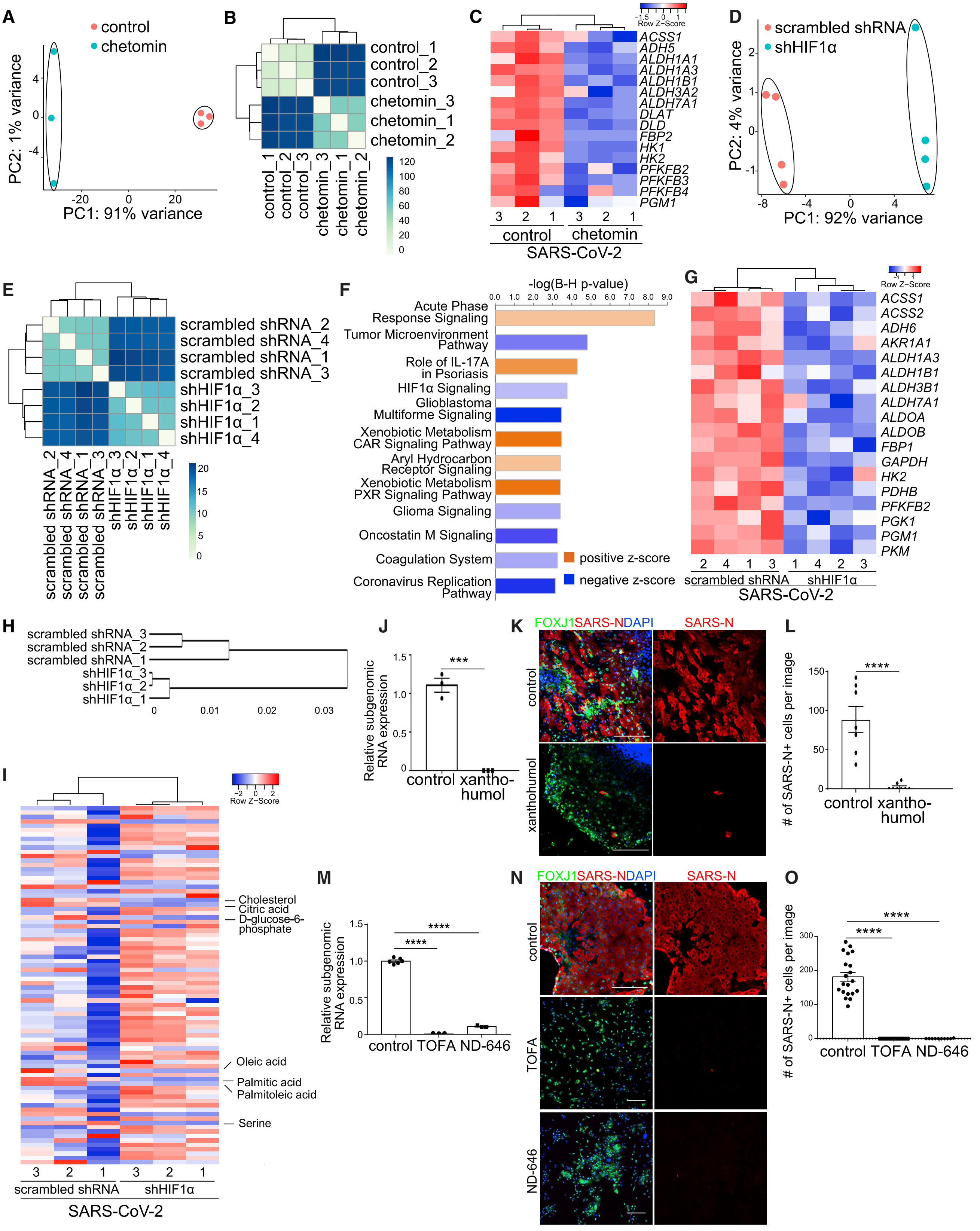

(legend on next page)

promote SARS-CoV-2 infection in human monocytes by regulating HIF1α-dependent glycolysis (Codo et al., 2020). Taken together, we demonstrated that inhibiting the HIF1α--glycolysis axis blocks SARS-CoV-2 infection, which highlights the potential to target this metabolic pathway for development of anti-viral drugs.

STAR★METHODS

Detailed methods are provided in the online version of this paper and include the following:

- KEY RESOURCES TABLE
- RESOURCE AVAILABILITY
  - Lead contact
  - Materials availability
  - Data and code availability
- EXPERIMENTAL MODEL AND SUBJECT DETAILS
  - Cell lines
  - Viruses
- METHOD DETAILS
  - Airway organoid differentiation
  - Colonic organoids differentiation
  - SARS-CoV-2 infections
  - Immunohistochemistry
  - qRT-PCR
  - High throughput chemical screening
  - SARS-CoV-2 pseudovirus
  - Viral particle stability assay
  - Sequencing and gene expression UMI counts matrix generation
  - Single cell RNA-seq data analysis
  - RNA-Seq on hPSC-AOs
  - RNA-Seq on hPSC-COs
  - Metabolic profiling
  - HIF1a knockdown in hPSC-AOs
- QUANTIFICATION AND STATISTICAL ANALYSIS

SUPPLEMENTAL INFORMATION

Supplemental information can be found online at https://doi.org/10.1016/j.celrep.2021.109920.

ACKNOWLEDGMENTS

This work was supported by the Department of Surgery, Weill Cornell Medicine (T.E., S.C.); Bill and Melinda Gates Foundation (S.C., T.E., R.E.S.) (R01 DK124463, DP3 DK111907-01, R01 DK116075-01A1, R01 DK119667-01A1 to S.C.); NCI (R01CA234614), NIAID (2R01AI107301), and NIDDK (R01DK121072 and 1RO3DK117252), Department of Medicine, Weill Cornell Medicine (R.E.S.); and the Jack Ma Foundation (D.D.H.). S.C. and R.E.S. are supported as Irma Hirschl Trust Research Award Scholars. The Stable Isotope and Metabolomics Core Facility of the Diabetes Research and Training Center (DRTC) of the Albert Einstein College of Medicine is supported by NIH/NCI grant P60DK020541. We are also very grateful for technical support and advice from Harold Skip Ralph of the Microscopy and Image Analysis Core at WCM.

AUTHOR CONTRIBUTIONS

S.C., T.E., D.H., R.E.S., and H.W. conceived and designed the experiments. X.D. and X.T. performed organoid differentiation, drug screening, imaging, and qRT-PCR analysis. M.N., P.W., and Y.H. performed SARS-CoV-2-related experiments. T.Z., W.Z., and J.Z. performed the RNA-seq, single-cell RNA (scRNA)-seq, and bioinformatics analyses. Y.Q. performed metabolic profiling and data analysis.

DECLARATION OF INTERESTS

R.E.S. is on the scientific advisory board of Miromatrix. T.R.E. and S.C. are the co-founders of OncoBeat.

Received: November 12, 2020
Revised: July 1, 2021
Accepted: October 11, 2021
Published: October 15, 2021

REFERENCES

Anders, S., Pyl, P.T., and Huber, W. (2015). HTSeq–a Python framework to work with high-throughput sequencing data. Bioinformatics *31*, 166–169.

Appelberg, S., Gupta, S., Svensson Akusjärvi, S., Ambikan, A.T., Mikaeloff, F., Saccon, E., Végvári, Á., Benfeitas, R., Sperk, M., Ståhlberg, M., et al. (2020). Dysregulation in Akt/mTOR/HIF-1 signaling identified by proteo-transcriptomics of SARS-CoV-2 infected cells. Emerg. Microbes Infect. *9*, 1748–1760.

Bakhache, W., Neyret, A., McKellar, J., Clop, C., Bernard, E., Weger-Lucarelli, J., and Briant, L. (2019). Fatty acid synthase and stearoyl-CoA desaturase-1 are conserved druggable cofactors of Old World Alphavirus genome replication. Antiviral Res. *172*, 104642.

Figure 6. HIF1α regulates glycolysis in SARS-CoV-2-infected hPSC-AOs

(A and B) PCA (A) and sample clustering (B) analysis of control- and 1 μM chetomin-treated hPSC-AOs at 48 hpi (MOI = 0.2). n = 3 biological replicates.
(C) Heatmap of genes encoding enzymes involved in the glycolytic pathway of control- or 1 μM chetomin-treated hPSC-AOs at 48 hpi (MOI = 0.2).
(D–F) PCA (D), sample clustering (E), and IPA (F) analysis of the hPSC-AOs expressing shHIF1α or scrambled shRNA at 48 hpi (MOI = 0.2). n = 3 biological replicates.
(G) Heatmap of genes encoding enzymes involved in the glycolytic pathway in the hPSC-AOs expressing shHIF1α or scrambled shRNA at 48 hpi (MOI = 0.2).
(H and I) Hierarchical clustering analysis (H) and heatmap (I) of metabolic profiles of the hPSC-AOs expressing shHIF1α or scrambled shRNA at 48 hpi (MOI = 0.2). n = 3 biological replicates.
(J) qRT-PCR analysis for viral N sgRNA at 48 hpi of hPSC-AOs treated with control or 10 μM xanthohumol (MOI = 0.2). Data are presented as mean ± SEM. n = 3 biological replicates.
(K and L) Representative confocal images (K) and quantification (L) of SARS-N$^+$ cells at 48 hpi of hPSC-AOs, which were treated with control or 10 μM xanthohumol (MOI = 0.2). Scale bar, 100 μm. Data are presented as mean ± SEM. n = 6 biological replicates.
(M) qRT-PCR analysis for viral N sgRNA at 48 hpi of hPSC-AOs treated with control or 3 μM TOFA or 3 μM ND-646 (MOI = 0.2). Data are presented as mean ± SEM. n = 3 biological replicates.
(N and O) Representative confocal images (N) and quantification (O) of SARS-N$^+$ cells at 48 hpi of hPSC-AOs, which were treated with control or 3 μM TOFA or 3 μM ND-646 (MOI = 0.2). Scale bar, 100 μm. Data are presented as mean ± SEM. n = 6 biological replicates.
Data are presented as mean ± SEM. The p values were calculated by unpaired two-tailed Student's t test. *p < 0.05 and ***p < 0.001.
See also Figure S4 and Table S4.

Berenguer, J., Ryan, P., Rodríguez-Baño, J., Jarrín, I., Carratalà, J., Pachón, J., Yllescas, M., Arriba, J.R., Muñoz, E.A., Divasson, P.G., et al. (2020). Characteristics and predictors of death among 4035 consecutively hospitalized patients with COVID-19 in Spain. Clin. Microbiol. Infect. *26*, 1525–1536.

Blanco-Melo, D., Nilsson-Payant, B.E., Liu, W.C., Uhl, S., Hoagland, D., Møller, R., Jordan, T.X., Oishi, K., Panis, M., Sachs, D., et al. (2020). Imbalanced Host Response to SARS-CoV-2 Drives Development of COVID-19. Cell *181*, 1036–1045.e9.

Bojkova, D., Klann, K., Koch, B., Widera, M., Krause, D., Ciesek, S., Cinatl, J., and Münch, C. (2020). Proteomics of SARS-CoV-2-infected host cells reveals therapy targets. Nature *583*, 469–472.

Butler, A., Hoffman, P., Smibert, P., Papalexi, E., and Satija, R. (2018). Integrating single-cell transcriptomic data across different conditions, technologies, and species. Nat. Biotechnol. *36*, 411–420.

Cantuti-Castelvetri, L., Ojha, R., Pedro, L.D., Djannatian, M., Franz, J., Kuivanen, S., van der Meer, F., Kallio, K., Kaya, T., Anastasina, M., et al. (2020). Neuropilin-1 facilitates SARS-CoV-2 cell entry and infectivity. Science *370*, 856–860.

Carette, J.E., Stuiver, M., Van Lent, J., Wellink, J., and Van Kammen, A. (2000). Cowpea mosaic virus infection induces a massive proliferation of endoplasmic reticulum but not Golgi membranes and is dependent on de novo membrane synthesis. J. Virol. *74*, 6556–6563.

Chen, Y.W., Huang, S.X., de Carvalho, A.L.R.T., Ho, S.H., Islam, M.N., Volpi, S., Notarangelo, L.D., Ciancanelli, M., Casanova, J.L., Bhattacharya, J., et al. (2017). A three-dimensional model of human lung development and disease from pluripotent stem cells. Nat. Cell Biol. *19*, 542–549.

Chen, F., Chen, J., Yang, L., Liu, J., Zhang, X., Zhang, Y., Tu, Q., Yin, D., Lin, D., Wong, P.P., et al. (2019a). Extracellular vesicle-packaged HIF-1α-stabilizing lncRNA from tumour-associated macrophages regulates aerobic glycolysis of breast cancer cells. Nat. Cell Biol. *21*, 498–510.

Chen, H.J., Poran, A., Unni, A.M., Huang, S.X., Elemento, O., Snoeck, H.W., and Varmus, H. (2019b). Generation of pulmonary neuroendocrine cells and SCLC-like tumors from human embryonic stem cells. J. Exp. Med. *216*, 674–687.

Cherry, S., Kunte, A., Wang, H., Coyne, C., Rawson, R.B., and Perrimon, N. (2006). COPI activity coupled with fatty acid biosynthesis is required for viral replication. PLoS Pathog. *2*, e102.

Chukkapalli, V., Heaton, N.S., and Randall, G. (2012). Lipids at the interface of virus-host interactions. Curr. Opin. Microbiol. *15*, 512–518.

Codo, A.C., Davanzo, G.G., Monteiro, L.B., de Souza, G.F., Muraro, S.P., Virgilio-da-Silva, J.V., Prodonoff, J.S., Carregari, V.C., de Biagi Junior, C.A.O., Crunfli, F., et al. (2020). Elevated Glucose Levels Favor SARS-CoV-2 Infection and Monocyte Response through a HIF-1α/Glycolysis-Dependent Axis. Cell Metab. *32*, 437–446.e5.

Daly, J.L., Simonetti, B., Klein, K., Chen, K.E., Williamson, M.K., Antón-Plágaro, C., Shoemark, D.K., Simón-Gracia, L., Bauer, M., Hollandi, R., et al. (2020). Neuropilin-1 is a host factor for SARS-CoV-2 infection. Science *370*, 861–865.

Dobin, A., Davis, C.A., Schlesinger, F., Drenkow, J., Zaleski, C., Jha, S., Batut, P., Chaisson, M., and Gingeras, T.R. (2013). STAR: ultrafast universal RNA-seq aligner. Bioinformatics *29*, 15–21.

Dye, B.R., Hill, D.R., Ferguson, M.A., Tsai, Y.H., Nagy, M.S., Dyal, R., Wells, J.M., Mayhew, C.N., Nattiv, R., Klein, O.D., et al. (2015). In vitro generation of human pluripotent stem cell derived lung organoids. eLife *4*, e05098.

Fang, K.Y., Cao, W.C., Xie, T.A., Lv, J., Chen, J.X., Cao, X.J., Li, Z.W., Deng, S.T., and Guo, X.G. (2021). Exploration and validation of related hub gene expression during SARS-CoV-2 infection of human bronchial organoids. Hum. Genomics *15*, 18.

Fulcher, M.L., and Randell, S.H. (2013). Human Nasal and Tracheo-Bronchial Respiratory Epithelial Cell Culture. In Epithelial Cell Culture Protocols, Second Edition, S.H. Randell and M.L. Fulcher, eds. (Humana Press), pp. 109–121.

Guinea, R., and Carrasco, L. (1990). Phospholipid biosynthesis and poliovirus genome replication, two coupled phenomena. EMBO J. *9*, 2011–2016.

Han, Y., Duan, X., Yang, L., Nilsson-Payant, B.E., Wang, P., Duan, F., Tang, X., Yaron, T.M., Zhang, T., Uhl, S., et al. (2021). Identification of SARS-CoV-2 inhibitors using lung and colonic organoids. Nature *589*, 270–275.

Hawkins, F., Kramer, P., Jacob, A., Driver, I., Thomas, D.C., McCauley, K.B., Skvir, N., Crane, A.M., Kurmann, A.A., Hollenberg, A.N., et al. (2017). Prospective isolation of NKX2-1-expressing human lung progenitors derived from pluripotent stem cells. J. Clin. Invest. *127*, 2277–2294.

Hawkins, F.J., Suzuki, S., Beermann, M.L., Barillà, C., Wang, R., Villacorta-Martin, C., Berical, A., Jean, J.C., Le Suer, J., Matte, T., et al. (2021). Derivation of Airway Basal Stem Cells from Human Pluripotent Stem Cells. Cell Stem Cell *28*, 79–95.e8.

Heaton, N.S., and Randall, G. (2011). Multifaceted roles for lipids in viral infection. Trends Microbiol. *19*, 368–375.

Herker, E., Harris, C., Hernandez, C., Carpentier, A., Kaehlcke, K., Rosenberg, A.R., Farese, R.V., Jr., and Ott, M. (2010). Efficient hepatitis C virus particle formation requires diacylglycerol acyltransferase-1. Nat. Med. *16*, 1295–1298.

Hoffmann, M., Kleine-Weber, H., Schroeder, S., Krüger, N., Herrler, T., Erichsen, S., Schiergens, T.S., Herrler, G., Wu, N.H., Nitsche, A., et al. (2020). SARS-CoV-2 Cell Entry Depends on ACE2 and TMPRSS2 and Is Blocked by a Clinically Proven Protease Inhibitor. Cell *181*, 271–280.e8.

Huang, S.X., Islam, M.N., O'Neill, J., Hu, Z., Yang, Y.G., Chen, Y.W., Mumau, M., Green, M.D., Vunjak-Novakovic, G., Bhattacharya, J., and Snoeck, H.W. (2014). Efficient generation of lung and airway epithelial cells from human pluripotent stem cells. Nat. Biotechnol. *32*, 84–91.

Huang, S.X., Green, M.D., de Carvalho, A.T., Mumau, M., Chen, Y.W., D'Souza, S.L., and Snoeck, H.W. (2015). The in vitro generation of lung and airway progenitor cells from human pluripotent stem cells. Nat. Protoc. *10*, 413–425.

Hurley, K., Ding, J., Villacorta-Martin, C., Herriges, M.J., Jacob, A., Vedaie, M., Alysandratos, K.D., Sun, Y.L., Lin, C., Werder, R.B., et al. (2020). Reconstructed Single-Cell Fate Trajectories Define Lineage Plasticity Windows during Differentiation of Human PSC-Derived Distal Lung Progenitors. Cell Stem Cell *26*, 593–608.e8.

Jacob, F., Pather, S.R., Huang, W.K., Zhang, F., Wong, S.Z.H., Zhou, H., Cubitt, B., Fan, W., Chen, C.Z., Xu, M., et al. (2020). Human Pluripotent Stem Cell-Derived Neural Cells and Brain Organoids Reveal SARS-CoV-2 Neurotropism Predominates in Choroid Plexus Epithelium. Cell Stem Cell *27*, 937–950.e9.

Katsura, H., Sontake, V., Tata, A., Kobayashi, Y., Edwards, C.E., Heaton, B.E., Konkimalla, A., Asakura, T., Mikami, Y., Fritch, E.J., et al. (2020). Human Lung Stem Cell-Based Alveolospheres Provide Insights into SARS-CoV-2-Mediated Interferon Responses and Pneumocyte Dysfunction. Cell Stem Cell *27*, 890–904.e8.

Kechin, A., Boyarskikh, U., Kel, A., and Filipenko, M. (2017). cutPrimers: A New Tool for Accurate Cutting of Primers from Reads of Targeted Next Generation Sequencing. J. Comput. Biol. *24*, 1138–1143.

Kersten, S. (2014). Integrated physiology and systems biology of PPARα. Mol. Metab. *3*, 354–371.

Kung, A.L., Zabludoff, S.D., France, D.S., Freedman, S.J., Tanner, E.A., Vieira, A., Cornell-Kennon, S., Lee, J., Wang, B., Wang, J., et al. (2004). Small molecule blockade of transcriptional coactivation of the hypoxia-inducible factor pathway. Cancer Cell *6*, 33–43.

Lamers, M.M., Beumer, J., van der Vaart, J., Knoops, K., Puschhof, J., Breugem, T.I., Ravelli, R.B.G., Paul van Schayck, J., Mykytyn, A.Z., Duimel, H.Q., et al. (2020). SARS-CoV-2 productively infects human gut enterocytes. Science *369*, 50–54.

Lamers, M.M., van der Vaart, J., Knoops, K., Riesebosch, S., Breugem, T.I., Mykytyn, A.Z., Beumer, J., Schipper, D., Bezstarosti, K., Koopman, C.D., et al. (2021). An organoid-derived bronchioalveolar model for SARS-CoV-2 infection of human alveolar type II-like cells. EMBO J. *40*, e105912.

Li, H., Handsaker, B., Wysoker, A., Fennell, T., Ruan, J., Homer, N., Marth, G., Abecasis, G., and Durbin, R.; 1000 Genome Project Data Processing Subgroup (2009). The Sequence Alignment/Map format and SAMtools. Bioinformatics *25*, 2078–2079.

Liu, J., Zhang, X., Chen, K., Cheng, Y., Liu, S., Xia, M., Chen, Y., Zhu, H., Li, Z., and Cao, X. (2019). CCR7 Chemokine Receptor-Inducible lnc-Dpf3 Restrains Dendritic Cell Migration by Inhibiting HIF-1α-Mediated Glycolysis. Immunity *50*, 600–615.e15.

Liu, L., Wang, P., Nair, M.S., Yu, J., Rapp, M., Wang, Q., Luo, Y., Chan, J.F., Sahi, V., Figueroa, A., et al. (2020). Potent neutralizing antibodies directed to multiple epitopes on SARS-CoV-2 spike. bioRxiv. https://doi.org/10.1101/2020.06.17.153486.

Lorizate, M., and Kräusslich, H.G. (2011). Role of lipids in virus replication. Cold Spring Harb. Perspect. Biol. *3*, a004820.

Love, M.I., Huber, W., and Anders, S. (2014). Moderated estimation of fold change and dispersion for RNA-seq data with DESeq2. Genome Biol. *15*, 550.

Lun, A.T.L., McCarthy, D.J., and Marioni, J.C. (2016). A step-by-step workflow for low-level analysis of single-cell RNA-seq data with Bioconductor. F1000Res. *5*, 2122.

McCauley, K.B., Hawkins, F., Serra, M., Thomas, D.C., Jacob, A., and Kotton, D.N. (2017). Efficient Derivation of Functional Human Airway Epithelium from Pluripotent Stem Cells via Temporal Regulation of Wnt Signaling. Cell Stem Cell *20*, 844–857.e6.

Miyata, S., Inoue, J., Shimizu, M., and Sato, R. (2015). Xanthohumol Improves Diet-induced Obesity and Fatty Liver by Suppressing Sterol Regulatory Element-binding Protein (SREBP) Activation. J. Biol. Chem. *290*, 20565–20579.

Monteil, V., Kwon, H., Prado, P., Hagelkrüys, A., Wimmer, R.A., Stahl, M., Leopoldi, A., Garreta, E., Hurtado Del Pozo, C., Prosper, F., et al. (2020). Inhibition of SARS-CoV-2 Infections in Engineered Human Tissues Using Clinical-Grade Soluble Human ACE2. Cell *181*, 905–913.e7.

Montoro, D.T., Haber, A.L., Biton, M., Vinarsky, V., Lin, B., Birket, S.E., Yuan, F., Chen, S., Leung, H.M., Villoria, J., et al. (2018). A revised airway epithelial hierarchy includes CFTR-expressing ionocytes. Nature *560*, 319–324.

Morris, D.R., Qu, Y., Agrawal, A., Garofalo, R.P., and Casola, A. (2020). HIF-1α Modulates Core Metabolism and Virus Replication in Primary Airway Epithelial Cells Infected with Respiratory Syncytial Virus. Viruses *12*, 1088.

Mou, H., Zhao, R., Sherwood, R., Ahfeldt, T., Lapey, A., Wain, J., Sicilian, L., Izvolsky, K., Musunuru, K., Cowan, C., and Rajagopal, J. (2012). Generation of multipotent lung and airway progenitors from mouse ESCs and patient-specific cystic fibrosis iPSCs. Cell Stem Cell *10*, 385–397.

Nasheri, N., Joyce, M., Rouleau, Y., Yang, P., Yao, S., Tyrrell, D.L., and Pezacki, J.P. (2013). Modulation of fatty acid synthase enzyme activity and expression during hepatitis C virus replication. Chem. Biol. *20*, 570–582.

Ou, X., Liu, Y., Lei, X., Li, P., Mi, D., Ren, L., Guo, L., Guo, R., Chen, T., Hu, J., et al. (2020). Characterization of spike glycoprotein of SARS-CoV-2 on virus entry and its immune cross-reactivity with SARS-CoV. Nat. Commun. *11*, 1620.

Parker, R.A., Kariya, T., Grisar, J.M., and Petrow, V. (1977). 5-(Tetradecyloxy)-2-furancarboxylic acid and related hypolipidemic fatty acid-like alkyloxyarylcarboxylic acids. J. Med. Chem. *20*, 781–791.

Pei, R., Feng, J., Zhang, Y., Sun, H., Li, L., Yang, X., He, J., Xiao, S., Xiong, J., Lin, Y., et al. (2021). Host metabolism dysregulation and cell tropism identification in human airway and alveolar organoids upon SARS-CoV-2 infection. Protein Cell *12*, 717–733.

Perez, L., Guinea, R., and Carrasco, L. (1991). Synthesis of Semliki Forest virus RNA requires continuous lipid synthesis. Virology *183*, 74–82.

Petrilli, C.M., Jones, S.A., Yang, J., Rajagopalan, H., O'Donnell, L., Chernyak, Y., Tobin, K.A., Cerfolio, R.J., Francois, F., and Horwitz, L.I. (2020). Factors associated with hospital admission and critical illness among 5279 people with coronavirus disease 2019 in New York City: prospective cohort study. BMJ *369*, m1966.

Qiu, Y., Moir, R., Willis, I., Beecher, C., Tsai, Y.H., Garrett, T.J., Yost, R.A., and Kurland, I.J. (2016). Isotopic Ratio Outlier Analysis of the S. cerevisiae Metabolome Using Accurate Mass Gas Chromatography/Time-of-Flight Mass Spectrometry: A New Method for Discovery. Anal. Chem. *88*, 2747–2754.

Ramani, A., Müller, L., Ostermann, P.N., Gabriel, E., Abida-Islam, P., Müller-Schiffmann, A., Mariappan, A., Goureau, O., Gruell, H., Walker, A., et al. (2020). SARS-CoV-2 targets neurons of 3D human brain organoids. EMBO J. *39*, e106230.

Salahudeen, A.A., Choi, S.S., Rustagi, A., Zhu, J., van Unen, V., de la O, S.M., Flynn, R.A., Margalef-Català, M., Santos, A.J.M., Ju, J., et al. (2020). Progenitor identification and SARS-CoV-2 infection in human distal lung organoids. Nature *588*, 670–675.

Samuel, R.M., Majd, H., Richter, M.N., Ghazizadeh, Z., Zekavat, S.M., Navickas, A., Ramirez, J.T., Asgharian, H., Simoneau, C.R., Bonser, L.R., et al. (2020). Androgen Signaling Regulates SARS-CoV-2 Receptor Levels and Is Associated with Severe COVID-19 Symptoms in Men. Cell Stem Cell *27*, 876–889.e12.

Shang, J., Wan, Y., Luo, C., Ye, G., Geng, Q., Auerbach, A., and Li, F. (2020). Cell entry mechanisms of SARS-CoV-2. Proc. Natl. Acad. Sci. USA *117*, 11727–11734.

Sharma, M., Sasvari, Z., and Nagy, P.D. (2010). Inhibition of sterol biosynthesis reduces tombusvirus replication in yeast and plants. J. Virol. *84*, 2270–2281.

Sharma, M., Sasvari, Z., and Nagy, P.D. (2011). Inhibition of phospholipid biosynthesis decreases the activity of the tombusvirus replicase and alters the subcellular localization of replication proteins. Virology *415*, 141–152.

Silvas, J.A., Jureka, A.S., Nicolini, A.M., Chvatal, S.A., and Basler, C.F. (2020). Inhibitors of VPS34 and lipid metabolism suppress SARS-CoV-2 replication. bioRxiv.

Stuart, T., Butler, A., Hoffman, P., Hafemeister, C., Papalexi, E., Mauck, W.M., 3rd, Hao, Y., Stoeckius, M., Smibert, P., and Satija, R. (2019). Comprehensive Integration of Single-Cell Data. Cell *177*, 1888–1902.e1821.

Svensson, R.U., Parker, S.J., Eichner, L.J., Kolar, M.J., Wallace, M., Brun, S.N., Lombardo, P.S., Van Nostrand, J.L., Hutchins, A., Vera, L., et al. (2016). Inhibition of acetyl-CoA carboxylase suppresses fatty acid synthesis and tumor growth of non-small-cell lung cancer in preclinical models. Nat. Med. *22*, 1108–1119.

Tiwari, S.K., Wang, S., Smith, D., Carlin, A.F., and Rana, T.M. (2021). Revealing Tissue-Specific SARS-CoV-2 Infection and Host Responses using Human Stem Cell-Derived Lung and Cerebral Organoids. Stem Cell Reports *16*, 437–445.

Travaglini, K.J., Nabhan, A.N., Penland, L., Sinha, R., Gillich, A., Sit, R.V., Chang, S., Conley, S.D., Mori, Y., Seita, J., et al. (2020). A molecular cell atlas of the human lung from single-cell RNA sequencing. Nature *587*, 619–625.

van den Brink, S.C., Sage, F., Vértesy, Á., Spanjaard, B., Peterson-Maduro, J., Baron, C.S., Robin, C., and van Oudenaarden, A. (2017). Single-cell sequencing reveals dissociation-induced gene expression in tissue subpopulations. Nat. Methods *14*, 935–936.

Xu, H.E., Stanley, T.B., Montana, V.G., Lambert, M.H., Shearer, B.G., Cobb, J.E., McKee, D.D., Galardi, C.M., Plunket, K.D., Nolte, R.T., et al. (2002). Structural basis for antagonist-mediated recruitment of nuclear co-repressors by PPARalpha. Nature *415*, 813–817.

Yang, L., Han, Y., Nilsson-Payant, B.E., Gupta, V., Wang, P., Duan, X., Tang, X., Zhu, J., Zhao, Z., Jaffré, F., et al. (2020). A Human Pluripotent Stem Cell-based Platform to Study SARS-CoV-2 Tropism and Model Virus Infection in Human Cells and Organoids. Cell Stem Cell *27*, 125–136.e7.

Yuan, J., Peng, G., Xiao, G., Yang, Z., Huang, J., Liu, Q., Yang, Z., and Liu, D. (2020). Xanthohumol suppresses glioblastoma via modulation of Hexokinase 2 -mediated glycolysis. J. Cancer *11*, 4047–4058.

Zhang, B.Z., Chu, H., Han, S., Shuai, H., Deng, J., Hu, Y.F., Gong, H.R., Lee, A.C., Zou, Z., Yau, T., et al. (2020). SARS-CoV-2 infects human neural progenitor cells and brain organoids. Cell Res. *30*, 928–931.

Zhao, X., Howell, K.A., He, S., Brannan, J.M., Wec, A.Z., Davidson, E., Turner, H.L., Chiang, C.I., Lei, L., Fels, J.M., et al. (2017). Immunization-Elicited Broadly Protective Antibody Reveals Ebolavirus Fusion Loop as a Site of Vulnerability. Cell *169*, 891–904.e15.

Zhao, B., Ni, C., Gao, R., Wang, Y., Yang, L., Wei, J., Lv, T., Liang, J., Zhang, Q., Xu, W., et al. (2020). Recapitulation of SARS-CoV-2 infection and cholangiocyte damage with human liver ductal organoids. Protein Cell *11*, 771–775.

Zhou, J., Li, C., Liu, X., Chiu, M.C., Zhao, X., Wang, D., Wei, Y., Lee, A., Zhang, A.J., Chu, H., et al. (2020). Infection of bat and human intestinal organoids by SARS-CoV-2. Nat. Med. *26*, 1077–1083.

Ziegler, C.G.K., Allon, S.J., Nyquist, S.K., Mbano, I.M., Miao, V.N., Tzouanas, C.N., Cao, Y., Yousif, A.S., Bals, J., Hauser, B.M., et al. (2020). SARS-CoV-2 Receptor ACE2 Is an Interferon-Stimulated Gene in Human Airway Epithelial Cells and Is Detected in Specific Cell Subsets across Tissues. Cell *181*, 1016–1035.e19.

CellPress
OPEN ACCESS

STAR★METHODS

KEY RESOURCES TABLE

REAGENT or RESOURCE	SOURCE	IDENTIFIER
Antibodies		
Human ACE-2 Antibody	R & D Systems	#AF933 RRID:AB_355722
CDX2	Biogenex	#MU392A-UC RRID:AB_2650531
Cytokeratin-20	Santa Cruz	#sc-56522 RRID:AB_831485
FOXJ1	Thermo Fisher Scientific	#14-9965-82 RRID:AB_1548835
Acetyl-α-Tubulin	Cell Signaling	#5335 RRID:AB_2798556
acetyl-alpha tubulin	Sigma-Aldrich	#MABT868 RRID:AB_2819178
MUC5AC	Thermo Fisher Scientific	#MA5-12178 RRID:AB_10978001
P63	Biocare	#CM 163 A RRID:AB_10582730
SARS-CoV/SARS-CoV-2 Nucleocapsid Antibody	Sino Biological	#40143-R001 RRID:AB_2827974
Ki67 Antibody (SP6)	THERMO FISHER	#RM-9106-S1 RRID:AB_2314700
Donkey anti-Mouse IgG (H+L) Highly Cross-Adsorbed Secondary Antibody, Alexa Fluor 488	Thermo Fisher Scientific	#A-21202 RRID:AB_141607
Donkey anti-Rabbit IgG (H+L) Secondary Antibody, Alexa Fluor 594 conjugate	Thermo Fisher Scientific	#A-21207 RRID:AB_141637
Donkey anti-Rabbit IgG (H+L) Secondary Antibody, Alexa Fluor 647 conjugate	Thermo Fisher Scientific	#A-31573 RRID:AB_2536183
Donkey anti-Goat IgG (H+L) Secondary Antibody, Alexa Fluor 647	Thermo Fisher Scientific	#A-31571 RRID:AB_162542
Donkey anti-Goat IgG (H+L) Cross-Adsorbed Secondary Antibody, Alexa Fluor 647	Thermo Fisher Scientific	#A-21447 RRID:AB_141844
DAPI	Santa Cruz	#sc-3598
Chemicals, peptides, and recombinant proteins		
Y-27632	MedchemExpress	#HY-10583
CHIR99021	Cayman Chemical	#13122
Retinoic acid	Sigma Aldrich	#R2625-500MG
LDN 193189 hydrochloride - DM 3189 hydrochloride	Axon Medchem	#Axon 1509
L-Ascorbic acid	Sigma Aldrich	#A4544-100G
SB431542	R&D Systems	#1614/50
Dorsomorphin dihydrochloride	R&D Systems	#3093/50
IWP2	R&D Systems	#3533/50
Dexamethasone	Sigma-Aldrich	#D4902
8-Bromo-cAMP	Sigma-Aldrich	#B5386
IBMX	Sigma-Aldrich	#I5879
A83-01	Tocris Bioscience	#2939
DMH1	Tocris Bioscience	#4126
GW6471	Selleck	#S2798
Xanthohumol	MedchemExpress	#HY-N1067
Chetomin	Sigma-Aldrich	#C9623
ND-646	MedchemExpress	#HY-101842
TOFA	MedchemExpress	#HY-101068
Activin A	R&D Systems	#338-AC-500/CF

(Continued on next page)

Continued

REAGENT or RESOURCE	SOURCE	IDENTIFIER
Recombinant Human FGF-10 Protein	Peprotech	#100-26-500UG
Recombinant Human bFGF Protein	Peprotech	#100-18B-500UG
Recombinant Human BMP-4 Protein	R & D Systems	#314-BP
Recombinant Human EGF	Peprotech	#AF-100-15-500UG
Recombinant Human BMP-2	Peprotech	#AF-120-02
Recombinant Human FGF-4	Peprotech	#AF-100-31
Deposited data		
scRNA-seq	This paper	GEO: GSE160231
RNA-seq	This paper	GEO: GSE160231
Metabolism Profiling	This paper	NMDR: ST001921
Experimental models: Cell lines		
hESC line H1	WiCell	0043
RUES2	WiCell	0013
293T	ATCC	#CRL-11268
Vero E6	ATCC	#CRL-1586
Software and algorithms		
Code for data analysis	This paper	zenodo: https://zenodo.org/record/5533014
Cell Ranger	10X Genomics	https://support.10xgenomics.com/single-cell-gene-expression/software/overview/welcome
Scran	Lun et al., 2016	https://bioconductor.org/packages/release/bioc/html/scran.html
Rstudio	Rstudio	https://www.rstudio.com/
Seurat R package v3.1.0	Butler et al., 2018	https://satijalab.org/seurat/
DAVID6.8	LHRI	https://david.ncifcrf.gov/home.jsp
Adobe illustrator CC2017	Adobe	https://www.adobe.com/products/illustrator.html
Graphpad Prism 6	Graphpad software	https://www.graphpad.com
ToppCell Atlas	Toppgene	https://toppgene.cchmc.org/
Other		
StemFlex	GIBCO Thermo Fisher	#A3349401
mTeSR1 Complete Kit	Stem Cell Technologies	#85850
F12	GIBCO Thermo Fisher	#31765035
Penicillin-Streptomycin (5,000 U/mL)	GIBCO Thermo Fisher	#15070063
IMDM	GIBCO Thermo Fisher	#21056023
GlutaMAX Supplement	Thermo Fisher Scientific	#35050079
Accutase	StemCell Technologies, Inc.	# 07920
ReleSR	StemCell Technologies, Inc.	# 05872
B27	Thermo Fisher Scientific	# A3582801
Matrigel	Corning	#354234
N2 supplement-B	Stem Cell Technologies	#7156
DMEM/F12	Thermo Fisher Scientific	# 10565018
Advanced DMEM/F-12	GIBCO Thermo Fisher	# 12-634-028
RPMI 1640	GIBCO Thermo Fisher	#10-040-CV
Monothioglycerol	Sigma Aldrich	#M6145
PneumaCult-Ex Plus Medium	StemCell Technologies, Inc.	#05040
PneumaCult-ALI Medium	StemCell Technologies, Inc.	#05001

CellPress
OPEN ACCESS

RESOURCE AVAILABILITY

Lead contact

Further information and requests for resources and reagents should be directed to and will be fulfilled by the Lead Contact, Shuibing Chen (shc2034@med.cornell.edu)

Materials availability

This study did not generate new unique reagents.

Data and code availability

- scRNA-seq and RNA-seq data are available from the GEO repository database: GSE160231. The metabolism profiling data is available from National metabolomics data repository: ST001921.
- The code for scRNA-seq analysis is available at: https://zenodo.org/record/5533014.
- Any additional information required to reanalyze the data reported in this paper is available from the lead contact upon request.

EXPERIMENTAL MODEL AND SUBJECT DETAILS

Cell lines

The H1 and RUES2 hESCs were purchased from WiCell Research Institute Inc. The hESC H1 line was grown and maintained on 1% matrigel (Corning) coated six-well plates in homemade mTesR1 medium at 37°C under the 5% CO2 culture conditions. RUES2 hESCs were grown and maintained on 1% Matrigel-coated six-well plates in StemFlex medium (GIBCO) at 37°C with 5% CO2 culture condition. The medium was changed daily. When hESCs reached ~90% confluence, the cells were passaged at 1:6-1:10 with ReLeSR (Stem Cell Technology).

293 T cells and Vero E6 cells were purchased from ATCC and maintained in DMEM (GIBCO) +10% FBS (GIBCO) at 37°C with 5% CO2 culture condition.

Viruses

SARS-CoV-2 variants, including USA-WA1/2020 and B.1.351, were obtained from the World Reference Center for Emerging Viruses and Arboviruses located at the University of Texas, Medical Branch via the CDC.

METHOD DETAILS

Airway organoid differentiation

hPSC-AOs were derived using a protocol slightly modified from previous studies (Huang et al., 2014; McCauley et al., 2017). For definitive endoderm (DE) differentiation, cells were passaged with Accutase (Innovative Cell Technology) at 1:2-1:3 for the first day. When achieving 80%–90% confluency, hESCs were treated with 3 μM CHIR99021 (CHIR, Sigma) and 100 ng/ml Activin A (R&D systems) in basal medium RPMI1640 (Cellgro) supplemented with 1X Pen-Strep (GIBCO) for 1 day, and changed to the basal medium containing 100 ng/ml Activin A and 2% FBS for the next 48 hours. To induce anterior foregut endoderm, the endoderm cells were cultured in complete serum free differentiation (cSFD) medium supplemented with 2 μM dorsomorphin dihydrochloride (R&D Systems) and 10 μM SB431542 (R&D Systems) for 24 hours, and then switched to 10 μM SB431542 and 1 μM IWP2 (R&D Systems) treatment for 24 hours. For induction of early stage lung progenitor cells (day 15), the anterior foregut endoderm was treated with 3 μM CHIR99021, 10 ng/ml human BMP4 (peprotech) and 50 nM all-trans retinoic acid (ATRA) in cSFD medium. At day 15, the lung progenitor cells were detached from 2D culture by trypsinization, mixed with matrigel, and replated to form 70 μL domes on 24-well plates. The 3D culture was maintained in cSFD containing 100 ng/ml human FGF10, 250 ng/ml human FGF2, 50 nM dexamethasone, 0.1 mM 8-bromo-cAMP (Sigma Aldrich) and 0.1 mM IBMX (Sigma Aldrich). To promote the formation of ciliated cells, the air-liquid culture system was adopted (Fulcher and Randell, 2013; Hawkins et al., 2021). D30 hPSC-AOs were switched to culturing in PneumaCult Ex Plus medium (StemCell Technologies, Inc.) supplemented with 1 μM A83-01(Tocris) and 1 μM DMH1(Tocris) for at least 10 days. At day 40, the hPSC-AOs above were dissociated into single cells and seeded into 0.4 μm transwell permeable support inserts coated with 1% Matrigel at 30,000 cells per insert to expand for another 10 days. The culture medium in both upper and lower chambers was changed to PneumaCult ALI medium (StemCell Technologies, Inc.) for inducing the formation of ciliated cells at day 49. The medium on the top was removed the next day. The cells were exposed to air for at least 14 days for further differentiation.

Colonic organoids differentiation

For definitive endoderm (DE) differentiation, cells were passaged with Accutase at 1:2-1:3 for the first day. When achieving 80%–90% confluency, hESCs were treated with 3 μM CHIR99021 and 100 ng/ml Activin A in basal medium RPMI1640 supplemented with 1X Pen-Strep for 1 day, and changed to the basal medium containing 100 ng/ml Activin A and 2% FBS for the next 48 hours.

To induce CDX2$^+$ hindgut endoderm, DE was treated with 3 μM CHIR99021 and 500 ng/ml FGF4 (Peprotech) in RPMI1640 supplemented with 1X B27 supplement (GIBCO) and 1X Pen-Strep (GIBCO) for 4 days with daily changing of fresh media. Spheroids began to bud out from the 2D culture during the hindgut differentiation process. The hindgut endoderm was then subjected to colonic lineage induction by treatment with 100 ng/ml 3 μM CHIR99021, 100 ng/ml BMP2 (Peprotech), and 100 ng/ml hEGF (Peprotech) in Advance DMEM F12 medium supplemented with 1X B27 supplement (GIBCO), 1X GlutaMax (GIBCO), 10 mM HEPES (GIBCO) and 1X Pen-Strep for 3 days with daily changing of fresh medium. After colonic fate induction, the colon progenitor spheroids were collected from the initial 2D cultures and embedded in a Matrigel dome in a 24-well plate. Differentiation to mature colonic cell types was achieved by culturing these colon progenitor spheroids in differentiation medium containing 600 nM LDN193189 (Axon), 3 μM CHIR99021 and 100 ng/ml hEGF in Advance DMEM F12 medium supplemented with 1X B27 supplement, 1X GlutaMax, 10 mM HEPES and 1X Pen-Strep. The differentiation medium was changed every 3 days for at least 40 days to achieve full colonic differentiation. The hPSC-COs were passaged and expanded every 10 – 14 days at 1:6 density. To passage hPSC-COs, the Matrigel domes were scrapped off the plate and resuspended in cold splitting media (Advance DMEM F12 medium supplemented with 1X GlutaMax, 10 mM HEPES and 1X Pen-Strep). The hPSC-COs were mechanically dislodged from the Matrigel dome and fragmented by pipetting in cold splitting media. The old Matrigel and splitting media were removed after pelleting cells and the organoids were resuspended in 100% Matrigel. 50 μL Matrigel containing fragmentized hPSC-COs were plated in one well of a pre-warmed 24-well plates.

SARS-CoV-2 infections

SARS-CoV-2 variants, including USA-WA1/2020 and B.1.351, were obtained from the World Reference Center for Emerging Viruses and Arboviruses located at the University of Texas, Medical Branch via the CDC. SARS-CoV-2 was propagated in Vero E6 cells (ATCC) in EMEM supplemented with 10% FCS, 1 mM Sodium Pyruvate and 10 mM HEPES as described previously (Liu et al., 2020).

hPSC-AOs or hPSC-COs were fragmented into small cell clusters and plated on 10% matrigel-coated plates. The infection was performed in the culture media at the indicated MOIs at 37°C. For pre-infection treatment experiments, hPSC-AOs or hPSC-COs were pretreated with DMSO (control), 10 μM GW6471, 10 μM xanthohumol, 1 μM chetomin, 3 μM ND-646 or 3 μM TOFA for 4 hours prior to infection. For post-infection treatment experiments, hPSC-AOs or hPSC-COs were treated with DMSO (control) or 10 μM GW6471 at 24 hpi. At the indicated hpi, cells were washed three times with PBS. For RNA analysis cells were lysed in TRIzol (Invitrogen). For immunofluorescence staining cells were fixed in 4% formaldehyde for 60 minutes at room temperature.

All work involving live SARS-CoV-2 was performed in the CDC/USDA-approved BSL-3 facility at the Aaron Diamond AIDS Research Center located at Columbia University.

Immunohistochemistry

For FOXJ1 and ACE2 staining, the hPSC-AOs were fixed in 4% paraformaldehyde and transferred to 30% sucrose, followed by embedding and freezing in O.C.T (Fisher Scientific, Pittsburgh, PA). The hPSC-AOs or hPSC-COs were fixed in 4% formaldehyde, followed with permeabilization in PBS containing 0.1% Triton X-100. For immunofluorescence, cells or tissue sections were immunostained with primary antibodies at 4°C overnight and secondary antibodies at room temperature for 1 hour. The information for primary antibodies and secondary antibodies are provided in Table S5. Nuclei were counterstained by DAPI.

qRT-PCR

Total RNA samples were prepared from organoids using TRIzol and the Direct-zol RNA Miniprep Plus kit (Zymo Research) according to the manufacturer's instructions. To quantify viral replication, measured by the accumulation of subgenomic N transcripts, one-step quantitative real-time PCR was performed using SuperScript III Platinum SYBR Green One-Step qRT-PCR Kit (Invitrogen) with primers specific for the *TRS-L* and *TRS-B* sites for the N gene as well as *ACTB* as an internal reference as described previously (Yang et al., 2020). Quantitative real-time PCR reactions were performed on an Applied Biosystems QuantStudio 6 Flex Real-Time PCR Instrument. Delta-delta-cycle threshold (ΔΔCT) was determined relative to the *ACTB* and mock infected /treated samples. Error bars indicate the standard deviation of the mean from three biological replicates. The sequences of primers/probes are provided in Table S6.

High throughput chemical screening

hPSC-AOs were dissociated using TrypLE for 10 min in a 37°C water bath and replated into 10% Matrigel-coated 384-well plates at 10,000 cells/50 μl medium/well. After overnight plating, compounds from a library containing signaling pathway regulators and FDA-approved drugs were added at 10 μM. The detailed chemical information is listed as Table S1. DMSO treatment was used as a negative control. hPSC-AOs were then infected with SARS-CoV-2 (MOI = 0.2). After 48 hpi, hPSC-AOs were fixed for Immunofluorescence assays using the ImageXpress^{MICRO} Automated High-Content Analysis System to determine the percentage of SARS-N$^+$ cells (infection rate).

$$Zscore = \frac{The\ infection\ rate - the\ average\ of\ the\ infection\ rate}{STDEV\ of\ the\ infection\ rate}$$

To calculate EC_{50}, the infection rate was normalized to the average of the infection rate under DMSO-treated conditions. The efficacy curves were calculated using Prism GrapdPad. The relative survival rate was calculated by normalizing the cell number to the average of cell numbers at control conditions.

SARS-CoV-2 pseudovirus

Recombinant Indiana VSV (rVSV) expressing SARS-CoV-2 spikes were generated as previously described (Zhao et al., 2017). HEK293T cells were grown to 80% confluency before transfection with pCMV3-SARS-CoV-2-spike (kindly provided by Dr. Peihui Wang, Shandong University, China) using FuGENE 6 (Promega). Cells were cultured overnight at 37°C with 5% CO2. The next day, medium was removed and VSV-G pseudo-typed ΔG-luciferase (G*ΔG-luciferase, Kerafast) was used to infect the cells in DMEM at an MOI of 3 for 1 hour before washing the cells with 1 × DPBS three times. DMEM supplemented with anti-VSV-G antibody (I1, mouse hybridoma supernatant from CRL-2700; ATCC) was added to the infected cells and they were cultured overnight as described previously (Liu et al., 2020). The next day, the supernatant was harvested and clarified by centrifugation at 300 g for 10 minutes and aliquots stored at −80°C.

hPSC-AOs were seeded in 384-well plates and treated with chemicals, SARS-CoV-2- pseudovirus was added at the indicated MOI = 1 and the plate centrifuged at 1200 g for 1 hour. Then, the organoids were cultured at 37°C with 5% CO_2. At 24 hpi, organoids were harvested for luciferase assays following the Luciferase Assay System protocol (E1501, Promega)

Viral particle stability assay

To determine whether chemical affects viral particle stability, a concentrated SARS-CoV-2 virus preparation was pre-incubated with chemical for 3 hours. After a 1000-fold dilution, the pre-treated SARS-CoV-2 was added to hPSC-AOs at a final MOI = 0.2 along with chemical or control. Under these conditions, we can separate the impact of chemical on viral particles rather than on host cells.

Sequencing and gene expression UMI counts matrix generation

The 10X Libraries were sequenced on the Illumina NovaSeq6000 sequencer with pair-end reads (28 bp for read 1 and 91 bp for read 2). The sequencing data were primarily analyzed by the 10X cellranger pipeline (v3.0.2) in two steps. In the first step, cellranger *mkfastq* demultiplexed samples and generated fastq files; and in the second step, cellranger *count* aligned fastq files to the 10X pre-built human reference (GRCh38 v3.0.0) and extracted gene expression UMI counts matrix.

Single cell RNA-seq data analysis

We filtered cells with less than 500 or more than 6000 genes detected, cells with less than 1000 or more than 30000 UMIs detected, as well as cells with mitochondrial gene content greater than 15%, and used the remaining 7170 cells for downstream analysis.

We normalized the gene expression UMI counts using a deconvolution strategy implemented by the R scran package (v.1.14.1). In particular, we pre-clustered cells using the *quickCluster* function; we computed size factor per cell within each cluster and rescaled the size factors by normalization between clusters using the *computeSumFactors* function; we normalized the UMI counts per cell by the size factors and took a logarithm transform using the *normalize* function.

We identified highly variable genes using the *FindVariableFeatures* function in the R Seurat package (v3.1.0) (Stuart et al., 2019), and selected the top 3000 variable genes after excluding mitochondrial genes, ribosomal genes and dissociation-related genes. The list of dissociation-related genes was originally built on mouse data (van den Brink et al., 2017); we converted them to human ortholog genes using Ensembl BioMart. We scaled the data using the *ScaleData* function and performed PCA on the top 3,000 variable genes using the *RunPCA* function. We ranked the PCs based on the percentage of variance explained by each component using the *ElbowPlot* function and selected the top 21 PCs for downstream visualization and clustering analysis.

We ran Uniform Manifold Approximation and Projection (UMAP) dimensional reduction using the *RunUMAP* function in the R Seurat package with the number of neighboring points setting to 35 and training epochs setting to 1000. We clustered cells into nine clusters by constructing a shared nearest neighbor graph and then grouping cells of similar transcriptome profiles using the *FindNeighbors* function and *FindClusters* function (resolution set to 0.5) in the R Seurat package. After reviewing the clusters, we merged them into five clusters representing ciliated-like cells, goblet-like cells, basal cells and two proliferating cell clusters, for further analysis. We identified marker genes for the merged five clusters using the *FindMarkers* function in the R Seurat package, and selected top 10 positive marker genes per cluster for heatmap plot using the R pheatmap package. We generated UMAP and violin plots highlighting expressions of selected genes using the R ggplot2 package.

To evaluate cell fates of hPSC-derived cells, we collected unique marker genes for fifteen reference cell types in human adult lung based on a recent study (Travaglini et al., 2020). We compared the marker genes identified in our five clusters and those in the fifteen reference cell types by calculating the fraction of overlapping marker genes. We performed z-score transformation on raw fraction values across reference cell types and generated the cell-type similarity heatmap plot using the R pheatmap package. We examined the expression of adult human ciliated and proximal ciliated cell marker genes in the hPSC-derived cells. In particular, we assigned each cell a score by comparing the average expression of these marker genes to that of a random set of background genes using the *AddModuleScore* function in the R Seurat package.

RNA-Seq on hPSC-AOs

Total RNA was extracted in TRIzol (Invitrogen) and DNase I treated using the Directzol RNA Miniprep kit (Zymo Research) according to the manufacturer's instructions. RNaseq libraries of polyadenylated RNA were prepared using the TruSeq RNA Library Prep Kit v2 (Illumina) or TruSeq Stranded mRNA Library Prep Kit (Illumina) according to the manufacturer's instructions. cDNA libraries were sequenced with pair-end 51 bps using an Illumina NovaSeq6000 platform. The resulting reads were checked for quality using FastQC v0.10.1(https://www.bioinformatics.babraham.ac.uk/projects/fastqc) and were trimmed for adaptor sequences and low quality bases using cutadapt v1.18(Kechin et al., 2017). In order to measure viral gene expression, the trimmed reads were aligned to the human reference genome (GRCh37) combined with SARS-CoV-2 genome (NC_045512.2) using STAR aligner v.2.5.2b (Dobin et al., 2013). Raw gene counts were quantified using HTSeq-count v0.11.2(Anders et al., 2015).

We observed large fractions of viral reads in several SARS-CoV-2 infected hPSC-AOs, and as a result, the human gene counts are significantly lower than those in the mock and treated samples. To make a fair comparison, we down-sampled the raw gene counts in the mock and treated samples, including Mock hPSC-AOs, GW6471 treated hPSC-AOs at 48 hpi (MOI = 0.2) and 1 μM chetomin treated hPSC-AOs at 48 hpi (MOI = 0.2), such that the human gene counts in these samples are comparable to the median human gene counts in the SARS-CoV-2 infected hPSC-AOs.

We performed differential expression analysis on gene counts using R DESeq2 package v1.26.0 (Love et al., 2014), and selected differentially expressed genes with adjusted p value less than 0.05 and absolute log2 fold change greater than 1 for pathway analysis using QIAGEN Ingenuity Pathways Analysis (IPA). We applied regularized logarithm transformation to the counts data and performed principle component analysis (PCA) using the *plotPCA* function in the DESeq2 package. We measured the sample-to-sample distances by applying the R *dist* function to the transpose of the transformed counts data, and performed a hierarchical clustering based on the distance using the R *hc* function. We generated heatmap plots based on the transformed counts data using Heatmapper. We generated volcano plots using the R ggplot2 package.

We calculated read coverage on the viral transcriptome by extracting the number of reads at each genomic position using *samtools* (Li et al., 2009) *coverage*. We normalized the coverage by the total number of aligned reads in each sample and plotted the coverage per million reads using R ggplot package.

RNA-Seq on hPSC-COs

cDNA libraries were sequenced with pair-end 51 bps using an Illumina NovaSeq6000 platform. The resulting reads were checked for quality using FastQC v0.10.1 and were trimmed for adaptor sequences and low quality bases using cutadapt v1.18 (Dobin et al., 2013) . In order to measure viral gene expression, the trimmed reads were aligned to the human reference genome (GRCh37) combined with SARS-CoV-2 genome (NC_045512.2) using STAR aligner v.2.5.2b. Raw gene counts were quantified using HTSeq-count v0.11.2(Anders et al., 2015).

We performed differential expression analysis on the gene counts using R DESeq2 (Love et al., 2014) package v1.26.0, and selected differentially expressed genes with adjusted p value less than 0.05 and absolute log2 fold change greater than 1 for pathway analysis using QIAGEN Ingenuity Pathways Analysis (IPA). We applied regularized logarithm transformation to the counts data and performed principle component analysis (PCA) using the *plotPCA* function in the DESeq2 package. We measured the sample-to-sample distances by applying the R *dist* function to the transpose of the transformed counts data, and performed a hierarchical clustering based on the distance using the R *hc* function. We generated volcano plots using the R ggplot2 package.

Metabolic profiling

hPSC-AOs were collected into 150 mM ammonium acetate. After centrifugation, cell pellets were collected for metabolite profiling. Metabolite extraction was performed with 200 μL of 80% methanol (20% water) with internal standards ($U^{13}C$ succinate, and $U^{13}C$ citrate, and heptadecanoate). The extracts were dried under gentle nitrogen flow. The dried samples were subjected to a two-step derivatization procedure (methoximation and silylation), and analyzed with GC-TOF/MS (GCT Premier, Waters Corporation) as described in our previous publication (Qiu et al., 2016).

In brief, the samples were methoximized with 50 μl of methoxyamine hydrochloride (MOA, 15 mg/mL in pyridine) at 30°C for 90 minutes. The silylation step was done with 50 μl of N,O-Bis(trimethylsilyl)trifluoroacetamide (BSTFA, containing 1% TMCS, Supelco, PA, USA) at 70°C for 60 minutes. After the samples cooled to room temperature, they were analyzed by GC-TOF/MS. Separation was performed with a 30-m DB-5MS column coupled with 10-m guard column. Helium was used as carrier gas at a flow of 1 ml/min. The oven program is as the following: started at 60°C for 1 minute, 10°C /min to 320 minutes and kept for 3 minutes.

Raw data files (.raw) generated from GC-TOF/MS were analyzed in Refiner MS (Genedata Expressionist, Basel, Switzerland) software. The output from Refiner MS contains retention time, quantification mass, and peak volume for each metabolite. Metabolite annotation was performed by searching against the mass spectrometry using our in-house library, Fiehn library and NIST 14 libraries in Refiner MS. To minimize the variations from cell number difference among different samples, the data was normalized to the sum of all the annotated metabolites. The normalized data was used for the statistical analysis.

HIF1a knockdown in hPSC-AOs

Two shRNAs against HIF1α and one scrambled negative control non-effective shRNA in lentiviral GFP vectors were purchased from OriGene company. The shRNA sequences are shown in Table S3. To generate lentivirus particles, shRNA vectors and lentivirus

packaging vectors were co-transfected into 293T cells. The day 15 lung progenitor cells were infected with the collected lentivirus (MOI = 0.5) with 8 μg/ml polybrene. To increase the infection efficiency, the cells were centrifuged at 2300 rpm for 1 hour at 30°C. At 24 hpi, the cells were selected with 1 μg/ml puromycin for an additional 72 hours. After selection, the cells were dissociated into single cells and seeded into 24 well plates at 300-400 cells/μl for 3D organoid differentiation.

QUANTIFICATION AND STATISTICAL ANALYSIS

N = 3 independent biological replicates were used for all experiments unless otherwise indicated. n.s. indicates a non-significant difference. *P*-values were calculated by unpaired two-tailed Student's t test unless otherwise indicated. *p < 0.05, **p < 0.01, ***p < 0.001 and ****p < 0.001.

Cell Reports, Volume *37*

Supplemental information

An airway organoid-based screen identifies a role for the HIF1α-glycolysis axis in SARS-CoV-2 infection

Xiaohua Duan, Xuming Tang, Manoj S. Nair, Tuo Zhang, Yunping Qiu, Wei Zhang, Pengfei Wang, Yaoxing Huang, Jenny Xiang, Hui Wang, Robert E. Schwartz, David D. Ho, Todd Evans, and Shuibing Chen

Figure S1

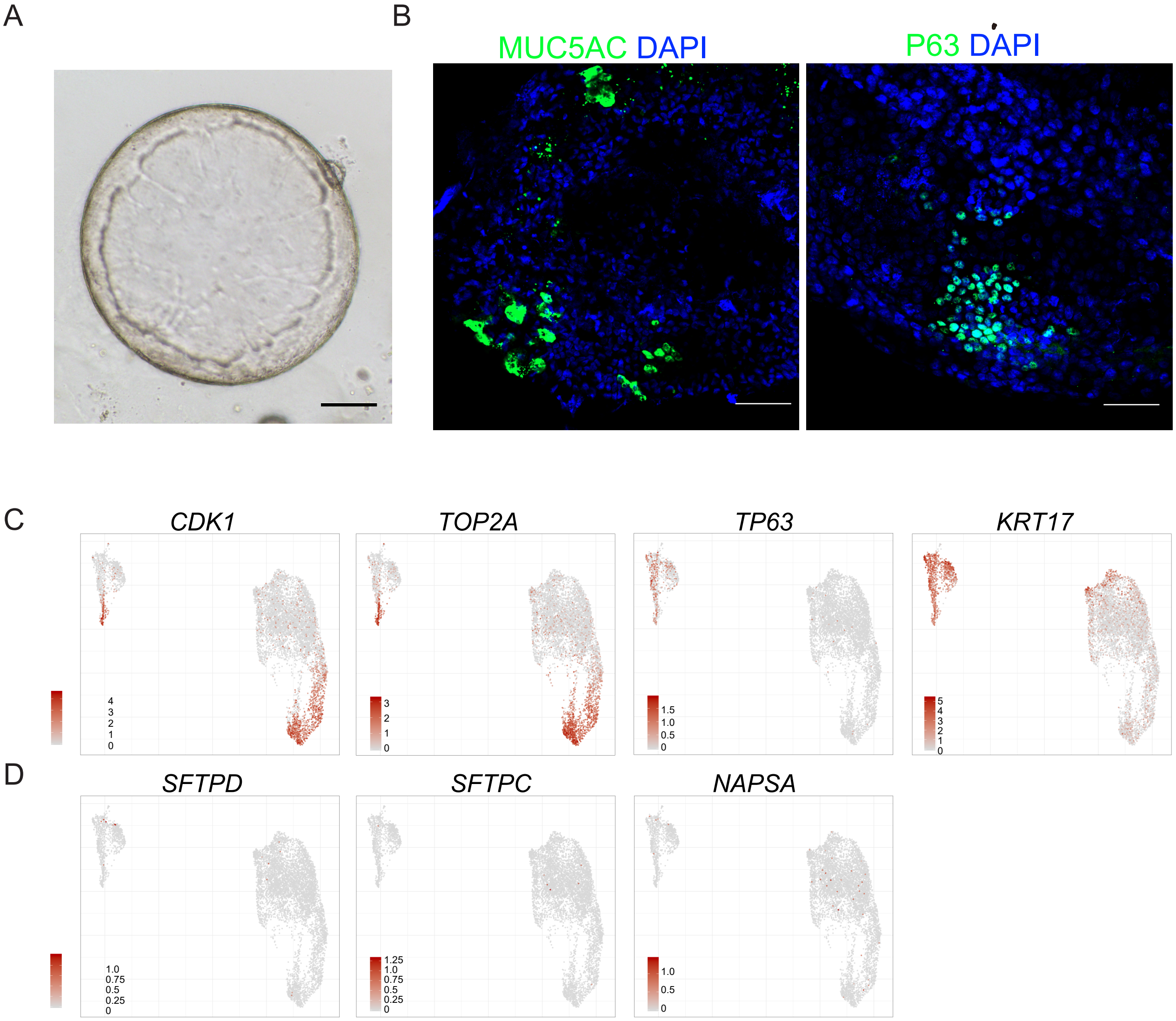
A
B
MUC5AC DAPI
P63 DAPI
C
CDK1
TOP2A
TP63
KRT17
D
SFTPD
SFTPC
NAPSA

Figure S1. Derivation and Characterization of hPSC-AOs.

(A) Phase contrast image of a representative hPSC-ASs. Scale bar = 400 μm.

(B) Representative confocal images of goblet cell marker MUC5AC and basal cell marker P63 in hPSC-AOs. DAPI stains nuclei. Scale bar = 100 μm.

(C and **D)** UMAP showing the expression levels of (C) hPSC-AOs cell markers and (D) alveolar epithelial type 2 cell markers**.** Related to Figure 1.

Figure S2

A

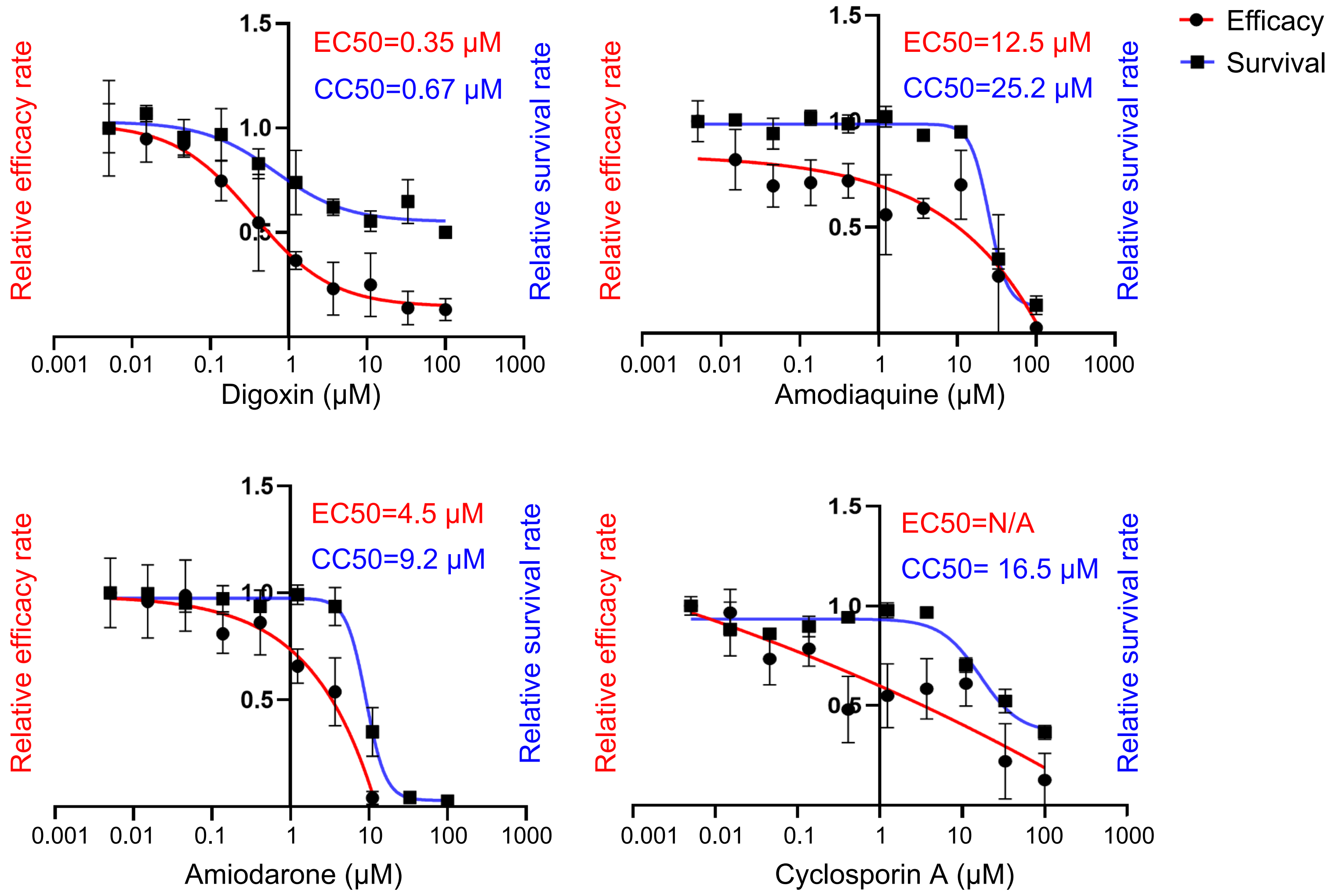
Efficacy
Survival
Relative efficacy rate
Relative survival rate
EC50=0.35 μM
CC50=0.67 μM
Digoxin (μM)
EC50=12.5 μM
CC50=25.2 μM
Amodiaquine (μM)
EC50=4.5 μM
CC50=9.2 μM
Amiodarone (μM)
EC50=N/A
CC50= 16.5 μM
Cyclosporin A (μM)
1.5
1.0
0.5
0.001
0.01
0.1
1
10
100
1000

B

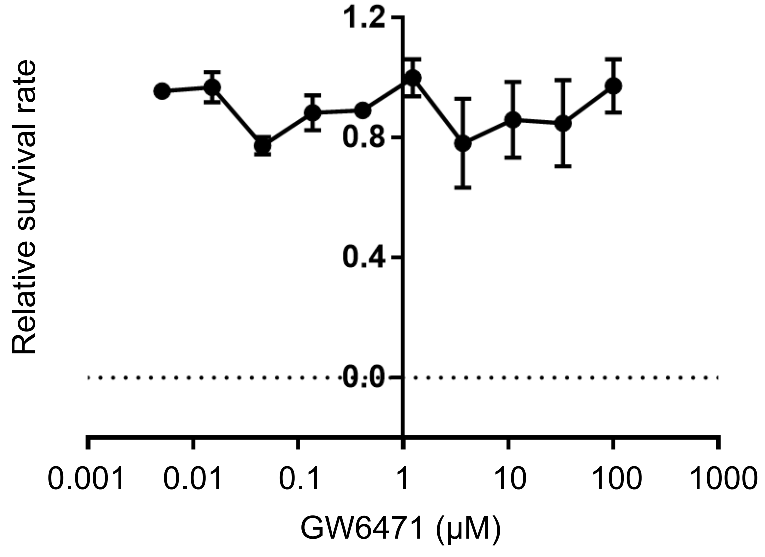
Relative survival rate
1.2
0.8
0.4
0.0
0.001
0.01
0.1
1
10
100
1000
GW6471 (μM)

Figure S2. Confirmation of hit compounds from high content screen.

(A) Efficacy and toxicity curves of hit compounds identified from the high content screen.

(B) Cell survival curve of GW6471 on hPSC-AOs.

Data is presented as mean ± SEM. N=3 biological replicates. Related to Figure 3.

Figure S3

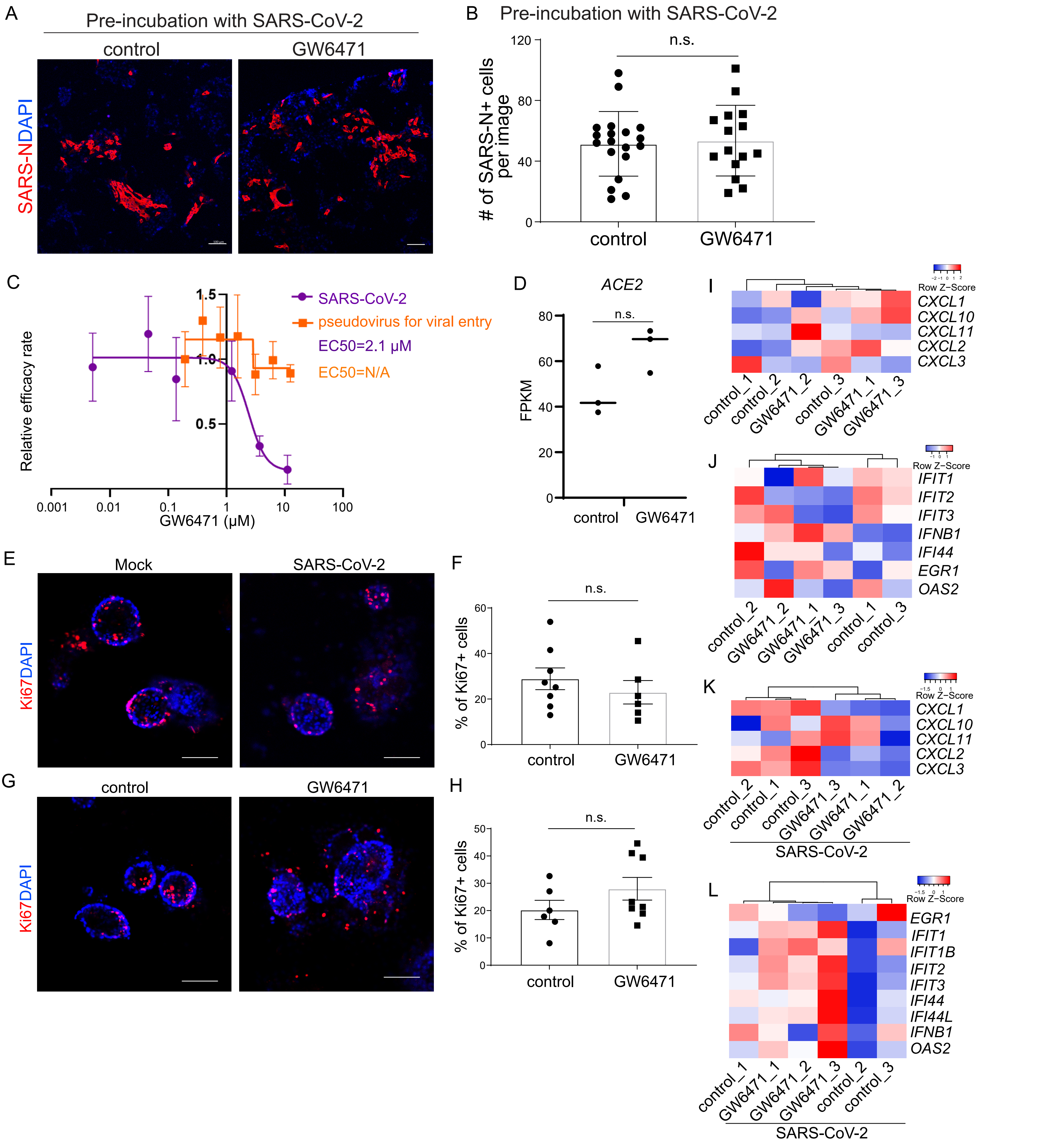
A
Pre-incubation with SARS-CoV-2
control
GW6471
SARS-NDAPI
B
Pre-incubation with SARS-CoV-2
n.s.
# of SARS-N+ cells per image
control
GW6471
C
SARS-CoV-2
pseudovirus for viral entry
EC50=2.1 μM
EC50=N/A
Relative efficacy rate
GW6471 (μM)
D
ACE2
n.s.
FPKM
control
GW6471
I
Row Z-Score
CXCL1
CXCL10
CXCL11
CXCL2
CXCL3
control_1
control_2
GW6471_2
control_3
GW6471_1
GW6471_3
J
Row Z-Score
IFIT1
IFIT2
IFIT3
IFNB1
IFI44
EGR1
OAS2
control_2
GW6471_2
GW6471_1
GW6471_3
control_1
control_3
E
Mock
SARS-CoV-2
Ki67DAPI
F
n.s.
% of Ki67+ cells
control
GW6471
K
Row Z-Score
CXCL1
CXCL10
CXCL11
CXCL2
CXCL3
control_2
control_1
control_3
GW6471_3
GW6471_1
GW6471_2
SARS-CoV-2
G
control
GW6471
Ki67DAPI
H
n.s.
% of Ki67+ cells
control
GW6471
L
Row Z-Score
EGR1
IFIT1
IFIT1B
IFIT2
IFIT3
IFI44
IFI44L
IFNB1
OAS2
control_1
GW6471_1
GW6471_2
GW6471_3
control_2
control_3
SARS-CoV-2

Figure S3. The impact of GW6471 on viral cycle and host AOs.

(A and **B)** Representative confocal images (A) and quantification (B) at 48 hpi of SARS-N^+ cells of hPSC-AOs, infected with SARS-CoV-2 which was pre-incubated with GW6471 or control (MOI=0.2). Scale bar = 100 μm. Data is presented as mean ± SEM. N=3 biological replicates.

(C) Efficacy curves of GW6471 on SARS-CoV-2 or pseudovirus. Data is presented as mean ± SD. N=3 biological replicates.

(D) FPKM value of ACE2 of control or 10 μM GW6471 treated hPSC-AOs at 48 hpi (MOI=0.2). Data is presented as mean ± SD. N=3 biological replicates.

(E and **F)** Representative confocal images (E) and quantification (F) of $Ki67^+$ cells of mock or SARS-CoV-2 infected (MOI=0.2) hPSC-AOs. Scale bar = 100 μm. Data is presented as mean ± SEM. N=3 biological replicates.

(G and **H)** Representative confocal images (G) and quantification (H) of $Ki67^+$ cells of GW6471 or control treated hPSC-AOs (MOI=0.2). Scale bar = 100 μm. Data is presented as mean ± SEM. N=3 biological replicates.

(I and **K)** Heatmap of chemokine (I) and interferon (K) expression of GW6471 or control treated hPSC-AOs in the absence of SARS-CoV-2.

(J and **L)** Heatmap of chemokine (J) and interferon (L) expression of GW6471 or control treated hPSC-AOs in the presence of SARS-CoV-2 (MOI=0.2).

P values were calculated by unpaired two-tailed Student’s t test.

Related to Figure 4.

Figure S4

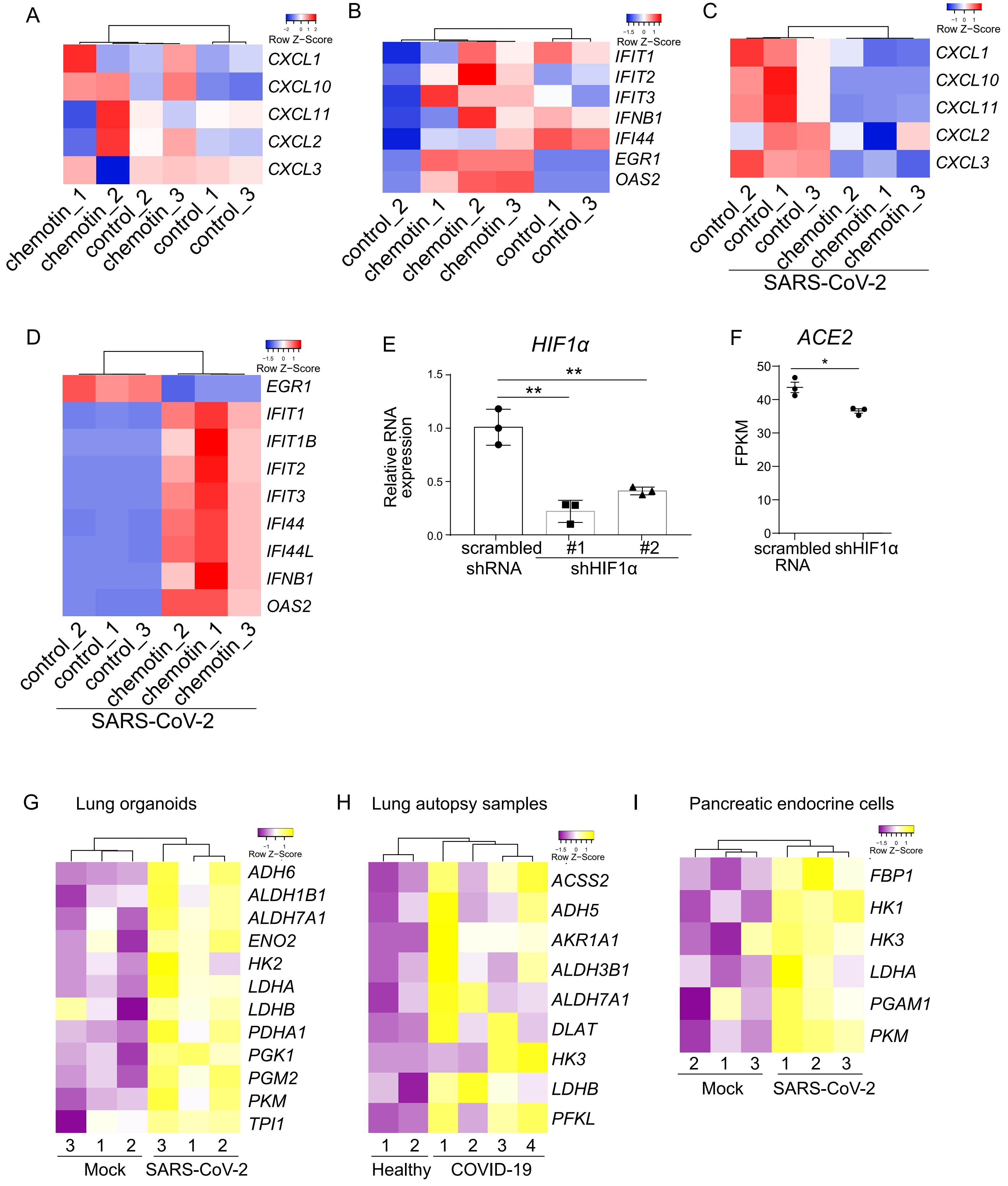
A
Row Z−Score
CXCL1
CXCL10
CXCL11
CXCL2
CXCL3
chemotin_1
chemotin_2
control_2
chemotin_3
control_1
control_3
B
Row Z−Score
IFIT1
IFIT2
IFIT3
IFNB1
IFI44
EGR1
OAS2
control_2
chemotin_1
chemotin_2
chemotin_3
control_1
control_3
C
Row Z−Score
CXCL1
CXCL10
CXCL11
CXCL2
CXCL3
control_2
control_1
control_3
chemotin_2
chemotin_1
chemotin_3
SARS-CoV-2
D
Row Z−Score
EGR1
IFIT1
IFIT1B
IFIT2
IFIT3
IFI44
IFI44L
IFNB1
OAS2
control_2
control_1
control_3
chemotin_2
chemotin_1
chemotin_3
SARS-CoV-2
E
HIF1α
Relative RNA expression
1.5
1.0
0.5
0.0
**
**
scrambled shRNA
#1
#2
shHIF1α
F
ACE2
FPKM
50
40
30
20
10
0
*
scrambled RNA
shHIF1α
G
Lung organoids
Row Z−Score
ADH6
ALDH1B1
ALDH7A1
ENO2
HK2
LDHA
LDHB
PDHA1
PGK1
PGM2
PKM
TPI1
3 1 2 3 1 2
Mock
SARS-CoV-2
H
Lung autopsy samples
Row Z−Score
ACSS2
ADH5
AKR1A1
ALDH3B1
ALDH7A1
DLAT
HK3
LDHB
PFKL
1 2 1 2 3 4
Healthy
COVID-19
I
Pancreatic endocrine cells
Row Z−Score
FBP1
HK1
HK3
LDHA
PGAM1
PKM
2 1 3 1 2 3
Mock
SARS-CoV-2

Figure S4. RNA-seq analysis.

(A and **B)** Heatmap of chemokine (A) and interferon (B) expression of chetomin or control treated hPSC-AOs in the absence of SARS-CoV-2.

(C and **D)** Heatmap of chemokine (C) and interferon (D) expression of chetomin or control treated hPSC-AOs in the presence of SARS-CoV-2 (MOI=0.2).

(E) Relative HIF1α mRNA expression in hPSC-AOs expressing shHIF1α or scrambled shRNA. Data is presented as mean ± SEM. N=3 biological replicates.

(F) FPKM value of ACE2 of control or chetomin-treated hPSC-AOs at 48 hpi (MOI=0.2). Data is presented as mean ± SD. N=3 biological replicates.

(**G**)Heatmap of glycolysis pathway associated genes in SARS-CoV-2 infected hPSC-lung organoids (GSE155241).

(**H**) Heatmap of glycolysis pathway associated genes in autopsy samples of COVID-19 patients (GSE155241).

(**I**) Heatmap of glycolysis pathway associated genes in SARS-CoV-2 infected pancreatic endocrine cells (GSE147903).

P values were calculated by unpaired two-tailed Student's t test. Related to Figure 4 and Figure 6.

Table S3. shRNAs for HIF1α knockdown. Related to Figure 4.

Scrambled shRNA	*GCACTACCAGAGCTAACTCAGATAGTACT*
shHIF1α_#1	*AGCTTGCTCATCAGTTGCCACTTCCACAT*
shHIF1α_#2	*TACGTTGTGAGTGGTATTATTCAGCACGA*

Table S5. Antibodies used for immunocytochemistry. Related to STAR*METHODS.

Usage	Antibody	Clone #	Host	Catalog #	Vendor	Dilution
Immunocytochemistry	Human ACE-2 Antibody	Polyclonal	Goat	AF933	R&D Systems	1:200
Immunocytochemistry	CDX2	CDX2-88	Mouse	MU392A-UC	Biogenex	1:500
Immunocytochemistry	Cytokeratin-20	SPM140	Mouse	sc-56522	Santa Cruz	1:100
Immunocytochemistry	FOXJ1	2A5	Mouse	14-9965-82	Thermo Fisher Scientific	1:100
Immunocytochemistry	acetyl-alpha tubulin	6-11B-1	Mouse	MABT868	Sigma-Aldrich	1:3000
Immunocytochemistry	acetyl-alpha tubulin	D20G3	Rabbit	5335	Cell Signaling	1:3000
Immunocytochemistry	MUC5AC	45M1	Mouse	MA5-12178	Thermo Fisher Scientific	1:100
Immunocytochemistry	P63	4A4	Mouse	CM163A	Biocare	1:100
Immunocytochemistry	SARS-CoV/SARS-CoV-2 Nucleocapsid Antibody	R001	Rabbit	40143-R001	Sino Biological	1:200
Immunocytochemistry	Ki67 Antibody	SP6	Rabbit	RM-9106-S1	Thermo Fisher Scientific	1:500
Immunocytochemistry	Donkey anti-Mouse IgG (H+L) Cross-Adsorbed Secondary Antibody, Alexa Fluor 488	Polyclonal	Donkey	#A-21202	Thermo Fisher Scientific	1:500

Immunocytochemistry	Donkey anti-Rabbit IgG (H+L) Secondary Antibody, Alexa Fluor 594	Polyclonal	Donkey	#A-21207	Thermo Fisher Scientific	1:500
Immunocytochemistry	Donkey anti-Goat IgG (H+L) Cross-Adsorbed Secondary Antibody, Alexa Fluor 647	Polyclonal	Donkey	#A-21447	Thermo Fisher Scientific	1:500
Immunocytochemistry	Donkey anti-Goat IgG Secondary Antibody, Alexa Fluor 594	Polyclonal	Donkey	A32816	Thermo Fisher	1:500
Immunocytochemistry	Donkey anti-Rabbit IgG Secondary Antibody, Alexa Fluor 647	Polyclonal	Donkey	A32795	Thermo Fisher	1:500

Table S6. Primers used for qRT-PCR. Related to STAR*METHODS.

**Primer name**	**Sequence**
ACTB-Forward	*CGTCACCAACTGGGACGACA*
ACTB-Reverse	*CTTCTCGCGGTTGGCCTTGG*
SARS-CoV-2-TRS-L	*CTCTTGTAGATCTGTTCTCTAAACGAAC*
SARS-CoV-2-TRS-N	*GGTCCACCAAACGTAATGCG*
HIF1α-Forward	*TATGAGCCAGAAGAACTTTTAGGC*
HIF1α-Reverse	*CACCTCTTTTGGCAAGCATCCTG*
